# Transcriptomic profiling of haloarchaeal denitrification through RNA-Seq analysis

**DOI:** 10.1128/aem.00571-24

**Published:** 2024-05-30

**Authors:** Jose María Miralles-Robledillo, Rosa María Martínez-Espinosa, Carmen Pire

**Affiliations:** 1 Biochemistry, Molecular Biology, Edaphology and Agricultural Chemistry Department, Faculty of Sciences, Universitat d'Alacant, Alicante, Spain; 2 Multidisciplinary Institute for Environmental Studies “Ramón Margalef”, University of Alicante, Alicante, Spain; Georgia Institute of Technology, Atlanta, Georgia, USA

**Keywords:** *Haloferax mediterranei*, haloarchaea, transcriptomics, denitrification, transcriptional regulation

## Abstract

Denitrification, a crucial biochemical pathway prevalent among haloarchaea in hypersaline ecosystems, has garnered considerable attention in recent years due to its ecological implications. Nevertheless, the underlying molecular mechanisms and genetic regulation governing this respiration/detoxification process in haloarchaea remain largely unexplored. In this study, RNA-sequencing was used to compare the transcriptomes of the haloarchaeon *Haloferax mediterranei* under oxic and denitrifying conditions, shedding light on the intricate metabolic alterations occurring within the cell, such as the accurate control of the metal homeostasis. Furthermore, the investigation identifies several genes encoding transcriptional regulators and potential accessory proteins with putative roles in denitrification. Among these are bacterioopsin-like transcriptional activators, proteins harboring a domain of unknown function (DUF2249), and cyanoglobin. In addition, the study delves into the genetic regulation of denitrification, finding a regulatory motif within promoter regions that activates numerous denitrification-related genes. This research serves as a starting point for future molecular biology studies in haloarchaea, offering a promising avenue to unravel the intricate mechanisms governing haloarchaeal denitrification, a pathway of paramount ecological importance.

**IMPORTANCE:**

Denitrification, a fundamental process within the nitrogen cycle, has been subject to extensive investigation due to its close association with anthropogenic activities, and its contribution to the global warming issue, mainly through the release of N_2_O emissions. Although our comprehension of denitrification and its implications is generally well established, most studies have been conducted in non-extreme environments with mesophilic microorganisms. Consequently, there is a significant knowledge gap concerning extremophilic denitrifiers, particularly those inhabiting hypersaline environments. The significance of this research was to delve into the process of haloarchaeal denitrification, utilizing the complete denitrifier haloarchaeon *Haloferax mediterranei* as a model organism. This research led to the analysis of the metabolic state of this microorganism under denitrifying conditions and the identification of regulatory signals and genes encoding proteins potentially involved in this pathway, serving as a valuable resource for future molecular studies.

## INTRODUCTION

Salty environments have been raising the interest of the scientific community for the last decades. Due to climate change and the progressive increase in salinity in some arid and semi-arid areas, the study of these ecosystems has become crucial (1–3). The most widespread studies on these extremophilic zones are concerned with understanding the composition of the population living in them, especially the microbial biodiversity, due to their potential interest for biotechnological purposes and their relevant roles in essential biogeochemical cycles (4–8). Nonetheless, information on the biological processes at the molecular level occurring in them is scarcer.

The nitrogen cycle is one of the most important biogeochemical cycles in nature. A great deal of effort has been put into understanding it, mainly because of their relationship with the efficiency of fertilization in soils used for agricultural purposes as well as with the emission of greenhouse gases such as nitrous and nitric oxide (1, 9, 10). Generally, hypersaline environments are dominated by haloarchaea, which are extremophilic microorganisms that belong to the *Halobacteria* class (*Archaea* domain) (6, 11, 12). These microorganisms are usually cataloged as partial denitrifiers, given the fact that they lack some genes of the complete denitrification pathway (1, 9). This translates into potential greenhouse gas emissions to the atmosphere in saline environments and therefore the study of the denitrification pathway in haloarchaea is an important topic for the scientific community (9).

*Haloferax mediterranei* is the model organism for denitrification studies in haloarchaea, as it is a complete denitrifier that can reduce nitrate to dinitrogen (13). The first studies with this microorganism deepened on the purification and biochemical characterization of the respiratory nitrate (14–16) and nitrite reductase (17, 18) and were followed by studies of the gas emission kinetics, transcription of the four genes that encode the main denitrification enzymes (*narG*, *nirK*, *nor,* and *nosZ*) and proteomics (13, 19, 20). These studies have shed light on some of the fundamental aspects of the denitrification pathway in haloarchaea, allowing the scientific community to understand better this pathway when driven by haloarchaea. However, the complete set of proteins in the electron transport chain and regulators that are involved in this process remained unknown until today. The only described transcriptional regulator is the oxygen sensor NarO identified in the haloarchaeon *Haloferax volcanii* (21). Recently, a proteomic approach was used to study the proteins that may be associated with denitrification in *H. mediterranei*, finding interesting proteins such as NarC that could be a component of the quinol oxidizing nitrate reductase complex, being the membrane attachment for NarGH. Also, the copper proteins, halocyanins, and azurins were associated as possible electron donors for NirK and NosZ, this being the first time that putative accessory elements of denitrification electron transport chains have been described in haloarchaea (20). These copper proteins are postulated to replace the role of c-type cytochromes, which traditionally act as electron donors in other microbial organisms (22). Another recent study identified the external signals that can activate this alternative respiration in *H. mediterranei* (23). In this study, the activation of the promoters of the genes that encode the main denitrification enzymes was analyzed and it was found that the promoter of the respiratory nitrate reductase is regulated differently from the promoters of the genes that encode the three other enzymes. In addition, regulatory motifs were identified in all the promoter regions, except for that of the nitrate reductase (23). Notably, the identity of the regulator capable of binding to this regulatory sequence remains undisclosed.

This lack of information on transcriptional regulators leaves a large knowledge gap in understanding this pathway. Due to this and to the advances in Next-Generation Sequencing (NGS), a comparative analysis of RNA-Seq in *H. mediterranei* was performed comparing oxic and denitrifying conditions. The differentially activated and suppressed pathways under denitrifying conditions have been analyzed, providing information about the processes that are changing in the cell metabolism of this haloarchaea when denitrification occurs. In addition, the differentially expressed regulators have been studied and discussed in this analysis together with other interesting proteins that could be related to the pathway under study.

This is the first detailed analysis of denitrification in haloarchaea, and it should be the starting point for future research in this field to decipher the regulatory machinery that controls the denitrification response in haloarchaea.

## RESULTS AND DISCUSSION

### Global and gene set enrichment analysis of *H. mediterranei* transcriptome under denitrifying conditions

**Fig 1:**
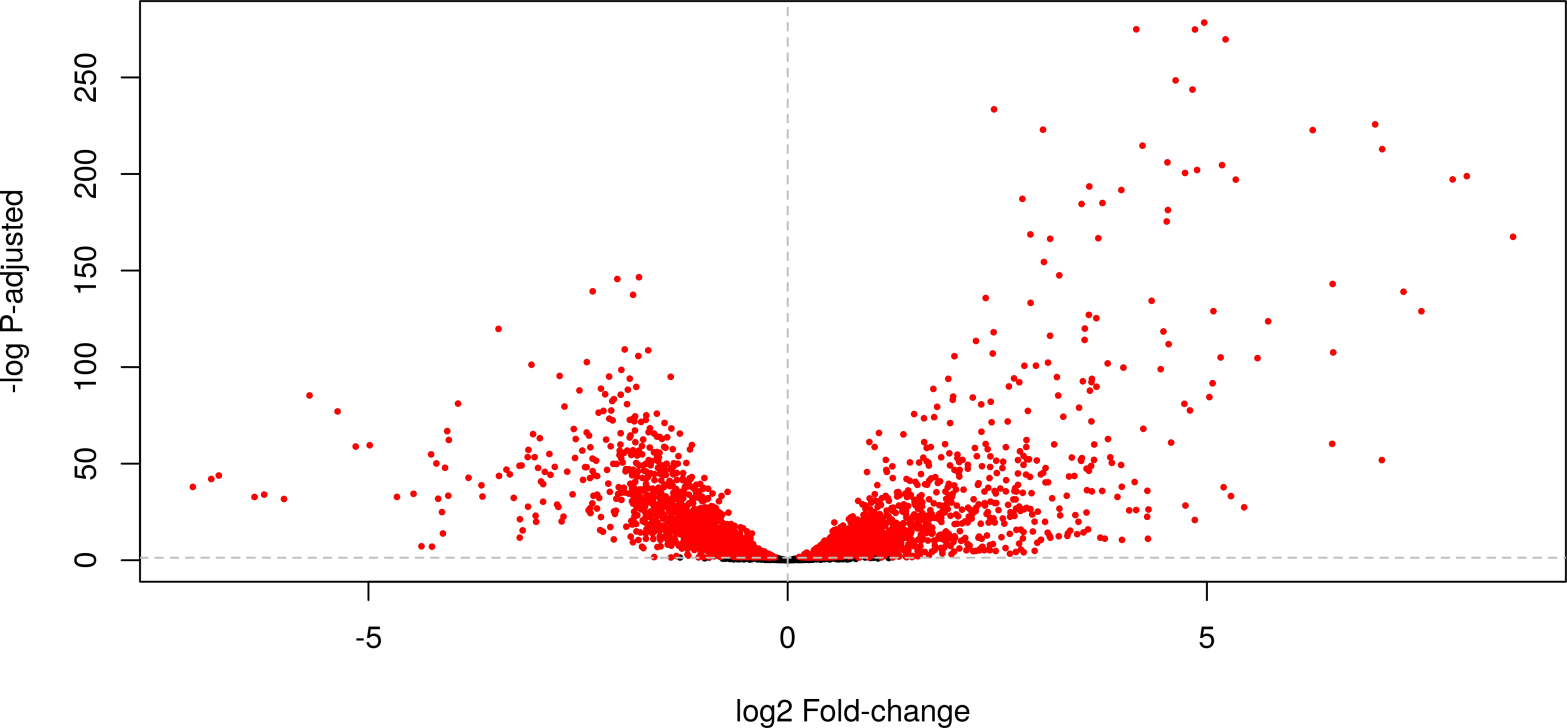
Volcano plot of the transcriptomic profile of differential gene expression between oxic and denitrifying conditions.

Denitrifying conditions imply a massive adaptation for *H. mediterranei* cells. The low oxygen concentration together with the need to reduce nitrate leads to important metabolic changes, in which *de novo* synthesis of some proteins and the repression of others requires a massive adjustment in transcription levels of different genes (20). Comparison between oxic and denitrifying conditions showed that among the 3,907 detected expressed genes (out of a total of 3,920 annotated genes), 416 were differentially expressed (log_2_FC < −2 or log_2_FC > +2 and padj <0.05). In all, 296 of these genes were upregulated under denitrifying conditions and 120 were underregulated (Fig. 1).

**Fig 2:**
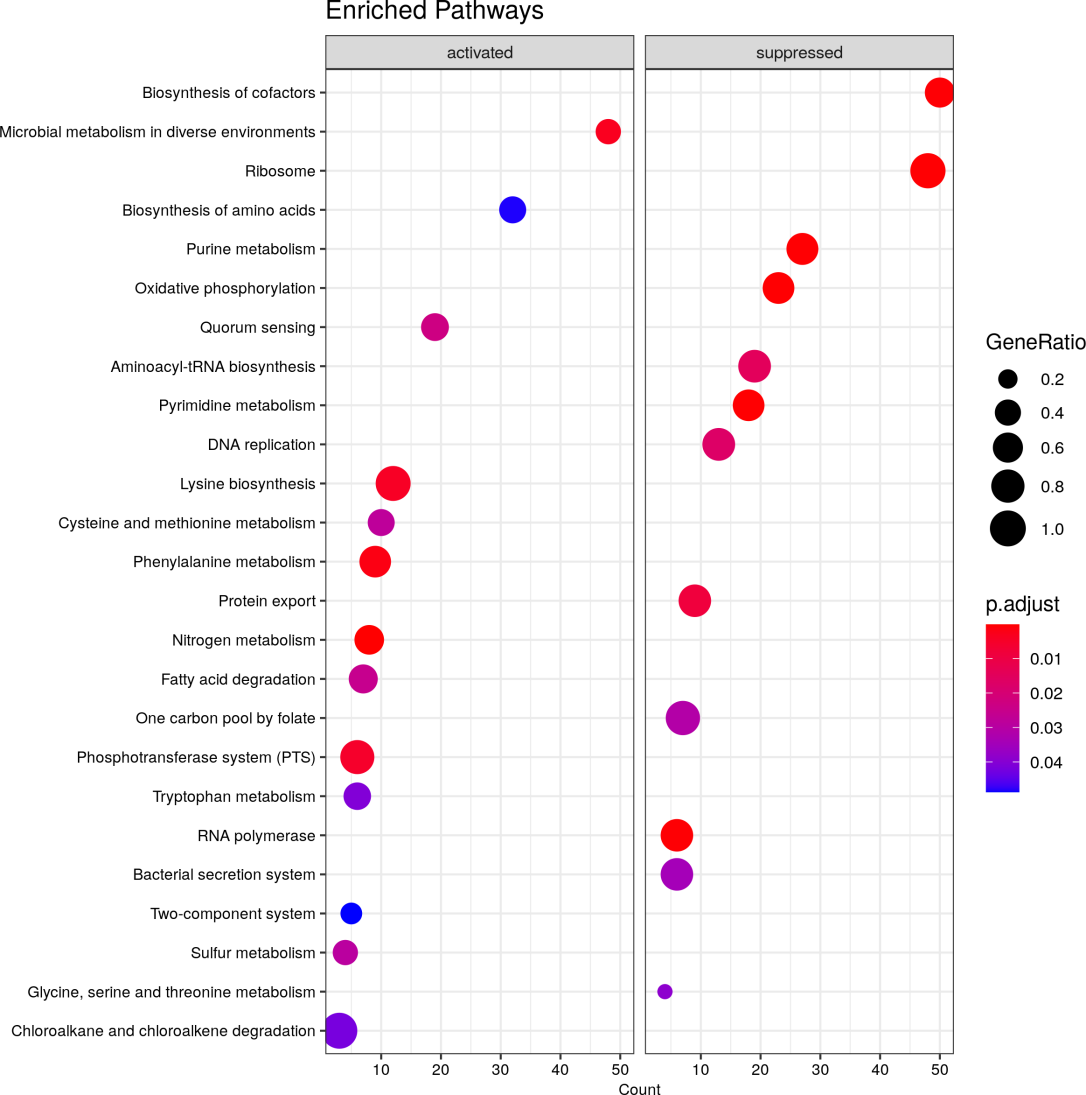
Gene set enrichment analysis (GSEA). Differentially suppressed or activated pathways of *H. mediterranei* under denitrifying conditions.

Analysis of differentially expressed genes by GSEA (Gene Set Enrichment Analysis) is usually done with Gene Ontology (GO) or KEGG databases (24–26). However, GO analysis was not possible with this data due to the lack of information about haloarchaea in this database. Only the KEGG database was used instead. KEGG showed the enriched pathways in the *H. mediterranei* transcriptome, considering the differentially expressed genes and the number of genes expressed in each pathway present. GSEA results are displayed in Fig. 2 and Table S1.

These results revealed that the statistically enriched pathways are associated with changes in the energy metabolism of *H. mediterranei*. Concerning the overall global changes, oxidative phosphorylation, replication, transcription, and translation are suppressed, which is consistent with the lower growth rates observed in *H. mediterranei* in previous studies under denitrifying conditions, where the energy yield is lower than in aerobic respiration (9, 27). On the other hand, nitrogen metabolism and fatty acid degradation were upregulated. In haloarchaea, fatty acid degradation is linked to the mobilization of polyhydroxyalkanoate (PHA) (28). *H. mediterranei* can produce PHA granules as carbon and energy storage (29). In fact, the E6P09_15675 and the E6P09_15680 genes encoding the PHA depolymerase PhaZh1 and the putative 3HB dehydrogenase BdhA showed a positive differential expression (log_2_FC = 3.62 and 3.66, respectively), and the genes encoding the main polymerase PhaEC (encoded by E6P09_17990 and E6P09_17995 genes) did not show significant changes (log_2_FC = 0.62 and 0.54, respectively). This may indicate that the cell could be using these reserves under denitrifying conditions since the function of PhaZh1 and BdhA in *H. mediterranei* is described as an *in vivo* PHA degradation pathway that produces acetoacetate as a final product (29).

Furthermore, the phosphotransferase system (PTS) was also activated. This system uses phosphoenolpyruvate as the phosphoryl donor to phosphorylate sugars for transport into the cell. In *H. mediterranei* the PTS gene cluster is located just adjacent to the *glpR-fruK* genes which encode the GlpR transcriptional regulator and the enzyme phosphofructokinase, which are also overexpressed in denitrifying conditions. The PTS gene cluster induction by fructose has been determined under the control of GlpR (30). The function of this system in the absence of fructose and denitrifying conditions is unclear. It could be related to the attempts of the cell to obtain energy or, as has been described in bacteria, it could not only function as a carbohydrate transporter but also regulate cellular processes by phosphorylating target proteins as transcriptional regulators (30).

Two-component system genes were also upregulated under this condition. This system usually allows the cell to sense and respond to the changes in the environment and has been related to denitrification in bacteria (31, 32). NarXL in bacteria is a two-component system, where NarX is a transmembrane sensor protein of nitrate and nitrite, whereas NarL is the protein that acts directly on the promoter regions of some denitrification-related genes (31, 32). Nevertheless, there are no homologous genes to *narX* and *narL* in *H. mediterranei* genome and there is no information about how *H. mediterranei* can sense the oxygen concentration and the presence of N-oxides, but it is worth exploring the possibility that these systems can have a role in it.

Moreover, it should be noted that quorum-sensing-related genes were also activated under this condition. Up to date, there are no reports about whether haloarchaeal denitrification is related to quorum sensing as in other organisms, but there is no doubt that these findings open new questions and ways to explore haloarchaeal denitrification (33, 34).

Intriguingly, the biosynthesis of cofactors was repressed, and this is an unexpected feature since several cofactors are needed for the new respiratory enzymes (Fe-S clusters, MoCo cofactor, etc.) (35). This repression could be due to an adaptation to an energetically less favorable situation in which non-essential biosynthetic pathways are repressed. However, the levels of repression are not high, and only three genes show a fold change greater than |−2|.

### Denitrification-related genes and more

**Fig 3:**
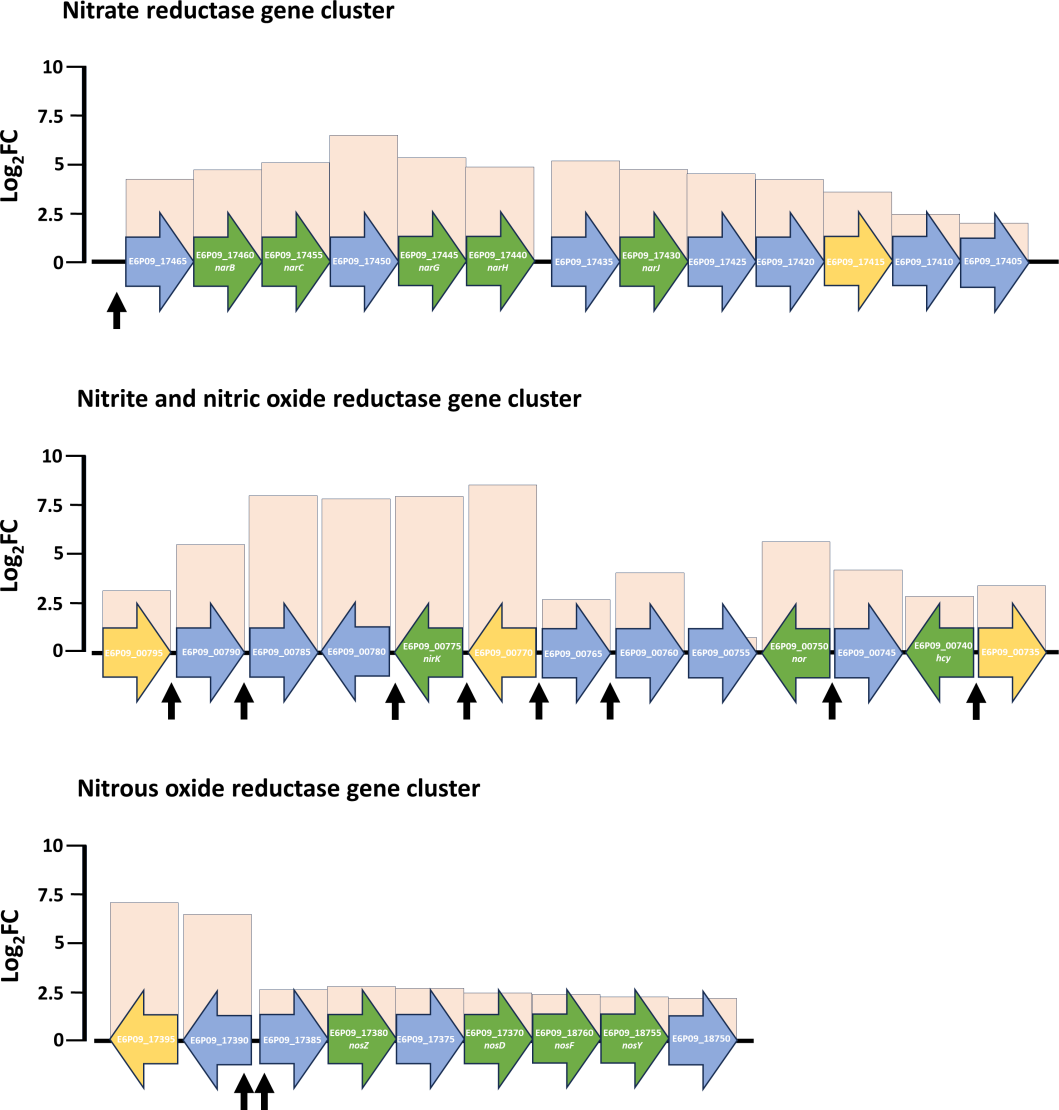
Gene clusters of the genes encoding the main enzymes of denitrification. Bars indicate the log_2_FC value of the genes (oxic *vs* denitrifying conditions). Green arrows indicate genes encoding the main denitrification enzymes or accessory proteins previously identified in other studies; orange arrows indicate the protein-coding genes discussed in this article in later sections; blue arrows indicate the rest of the genes. Small black arrows indicate promoter regions that present the regulatory motif found in different promoter regions (CGAAYATDKTYG) (further discussed in the next sections).

RNAseq analysis revealed that the transcription of operons and genes encoding the main denitrification enzymes as well as some previously identified accessory proteins (electron donors) were induced under denitrifying conditions versus oxic conditions (Fig. 3; Tables S2 to S4) (20). Intriguingly, other proteins that have not previously been linked with denitrification in *H. mediterranei* showed an increase in their transcription levels. These are DUF2249 proteins and cyanoglobin which could have a potential role in haloarchaeal denitrification.

*DUF2249 domain-containing proteins:* DUF2249 (Domain of unknown function 2249) is a protein domain widespread among proteins from all domains of life. Its function is unknown and the information about it is scarce, but this domain has been related to two different protein superfamilies. Proteins of these superfamilies present the DDUF2249 domain, or the gene encoding these enzymes is close to other genes encoding DUF2249-containing proteins in the genome (36, 37). DUF2249 has been related to the hemerythrin-like domain superfamily, which is a family mainly characterized by its ability to reversibly bind oxygen through its binuclear nonheme iron centers (37). However, this family is also involved in other interesting processes such as the repair of iron centers, the reduction of nitric oxide to nitrous oxide, or the binding of cations (37). DUF2249 has also been associated with the heme-copper oxidase superfamily (36). These enzymes are principally involved in the last step of aerobic respiration, reducing dioxygen to water. However, the nitric oxide reductases also belong to this superfamily and other heme-copper oxidases have been related to detoxification and exporting of N-compounds, maintenance of cellular iron homeostasis, and being part of the electron transference in denitrification (36). Moreover, two DUF2249-containing proteins from *Thermus thermophilus* called DrpA and DrpB have been linked directly with denitrification, being proposed as nitrate sensors (38).

The genome of *H. mediterranei* encodes four DUF2249-containing proteins, all of them differentially activated under denitrifying conditions. E6P09_00795 and E6P09_00770 genes (encoding one DUF2249 domain-containing protein each) are close to *nirK* and *nor* gene positions, E6P09_17395 (encoding one DUF2249 domain-containing protein) is between the *nar* and *nos* operons and E6P09_17415 is located in the *nar* operon (Fig. 3; Table 1; Table S2 to S4).

**TABLE 1:** Expression changes of all the DUF2249-containing proteins encoded in the *H. mediterranei* genome under denitrifying conditions (oxic *vs* denitrifying conditions)

NCBI locus tag	Gene product	log_2_FC
E6P09_00795	DUF2249 domain-containing protein	3.24
E6P09_00770	DUF2249 domain-containing protein	8.65
E6P09_17395	DUF2249 domain-containing protein	7.09
E6P09_17415	P-loop NTPase, iron-sulfur carrier protein (containing a DUF2249)	3.60

**Fig 4:**
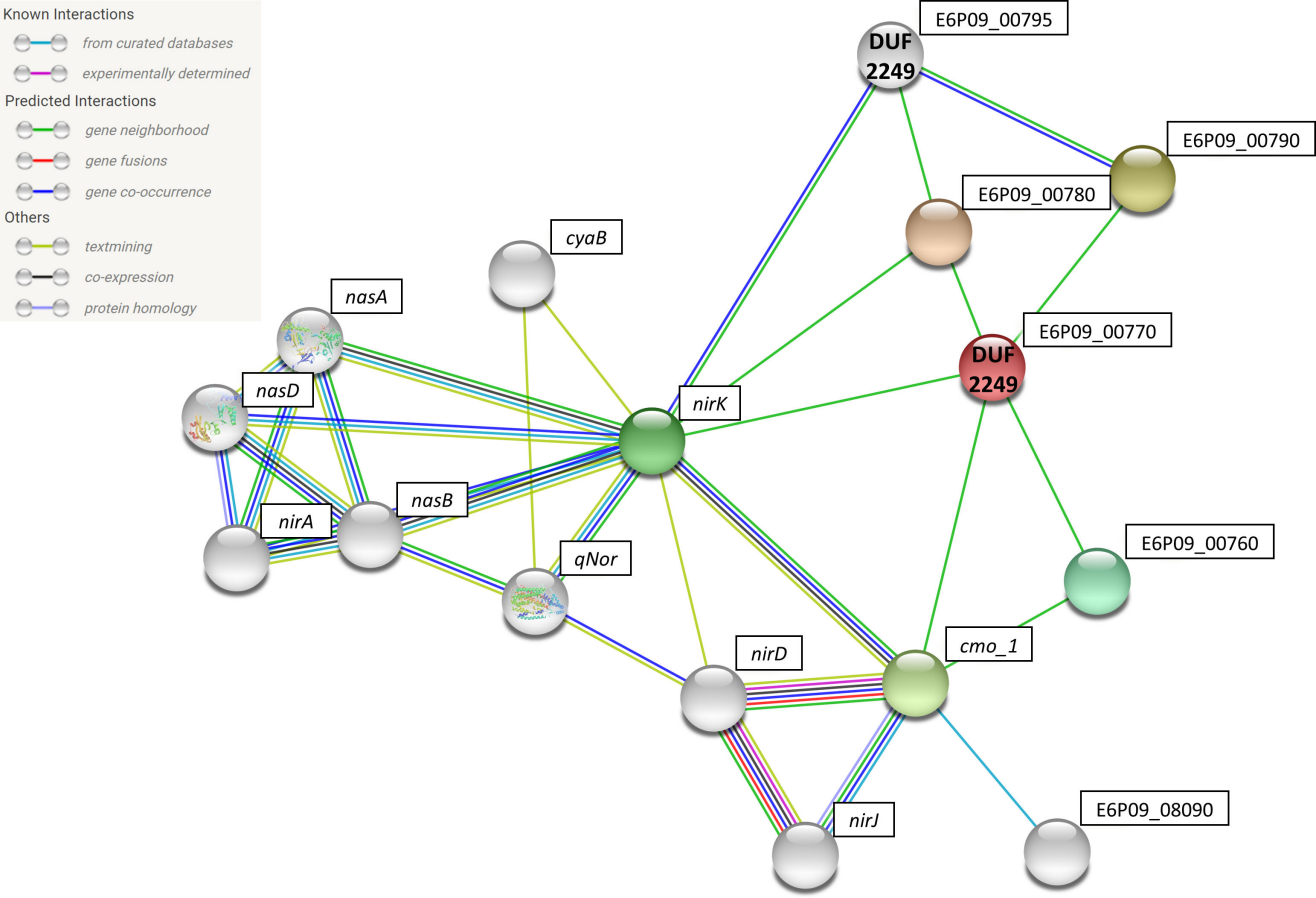
STRING network of the protein interaction of two DUF2249 related with nitrate metabolism enzymes.

E6P09_00795 and E6P09_00770 were analyzed by STRING to look for possible interactions with other proteins (Fig. 4) (E6P09_17395 and E6P09_17415 STRING analysis were not possible because the proteins encoded by these genes did not appear in the STRING database). STRING network showed that these proteins present direct interactions with *nirK* and genes that encode proteins related to electron transference, such as halocyanins (E6P09_00760), and to the biosynthesis of the PQQ coenzyme (*cmo*_1). Indirectly, they also present interaction with *nor,* assimilatory nitrate reductase (*nas*), assimilatory nitrite reductase (*nirA),* and proteins involved in archaeal heme biosynthesis (*nirD* and *nirJ*) (39, 40). Regarding this data, it seems that the DUF2249-containing proteins could have a direct or indirect role in the nitrate metabolism in *H. mediterranei*.

**Fig 5:**
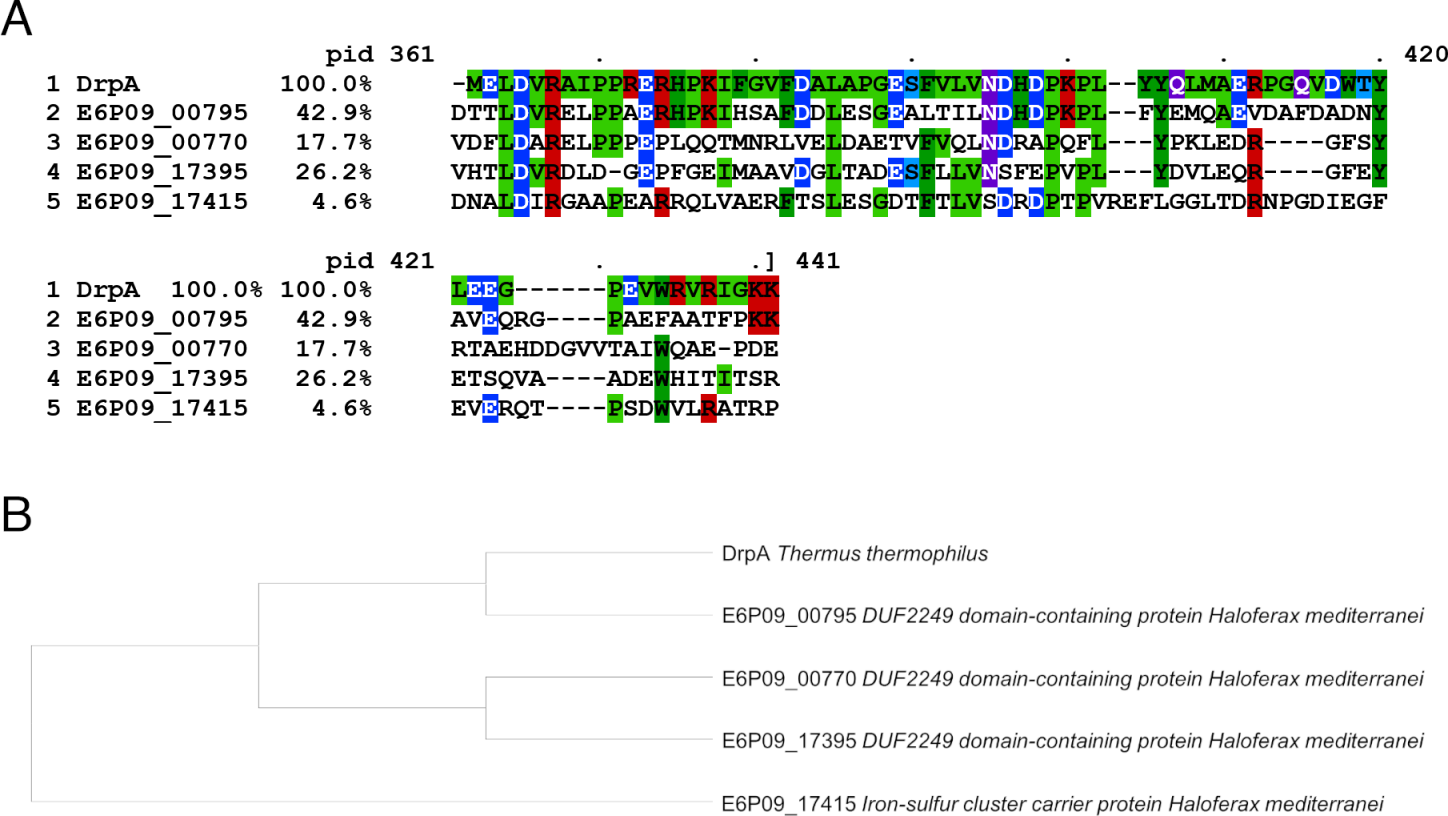
Comparisons of DUF2249 proteins. (**A**) Sequence alignment of all the DUF2249 domain-containing proteins present in the *H. mediterranei* genome together with DrpA from *T. thermophilus*. Colors indicate conserved amino acids. pid, percent identity compared to DrpA. (**B**) Phylogenetic tree of the DUF2249 domain-containing proteins. DrpA has the closest relationship with E6P09_00795 protein from *H. mediterranei*.

To deepen this topic, an alignment and a phylogenetic tree were built using the nitrate sensor DrpA from *T. thermophilus* and the *H. mediterranei* proteins (Fig. 5) (38). The alignment showed that E6P09_17415 presents differences in length with the other proteins because it also carries other domains apart from the DUF2249 (perhaps due to a gene fusion) (Fig. 5A). E6P09_00795 gene product is the most similar (42.9% of identity) to DrpA and is the only one that conserves the histidine residues that are present in DrpA and other homologs found in nitrate respiration gene clusters of different *Thermus* spp. and other genera (Fig. 5A and B) (38). Hence, this gene product could have a sensor function like DrpA.

*Cyanoglobin:* The gene E6P09_11805 of *H. mediterranei* encodes a cyanoglobin whose expression is increased under denitrifying conditions (log_2_FC = 4.16). Hemoglobins are well-known proteins because they are essential for oxygen transport (41). In the three domains of life, there are different types of hemoglobins, cyanoglobins being one of them (42, 43). Specifically, these belong to the group of truncated hemoglobins that have shown high binding capacities for oxygen (43, 44). However, this is not the only ligand they can bind, they also can bind other gases such as nitric oxide with high affinity (43, 44). This capacity of nitric oxide binding is related in some truncated hemoglobins with the potential dioxygenase activity that is present in some globins, which can efficiently convert nitric oxide to nitrate as a protective mechanism against nitrosative stress under conditions of low oxygen (43, 45, 46). Another function of globins is oxygen signaling, but this activity is not present in truncated hemoglobins like cyanoglobin (47). However, any of these functions has been proven in archaeal globins.

Reviewing, the exploration of the potential connection of DUF2249-containing proteins and cyanoglobins with denitrification showed interesting results that are very promising. Further investigation of these proteins using a genetic manipulation approach to obtain deletion/overexpression mutant strains would greatly help in understanding the role of these proteins in haloarchaeal denitrification if any.

### Transcriptional regulators with differential expression under denitrifying conditions

**Fig 6:**
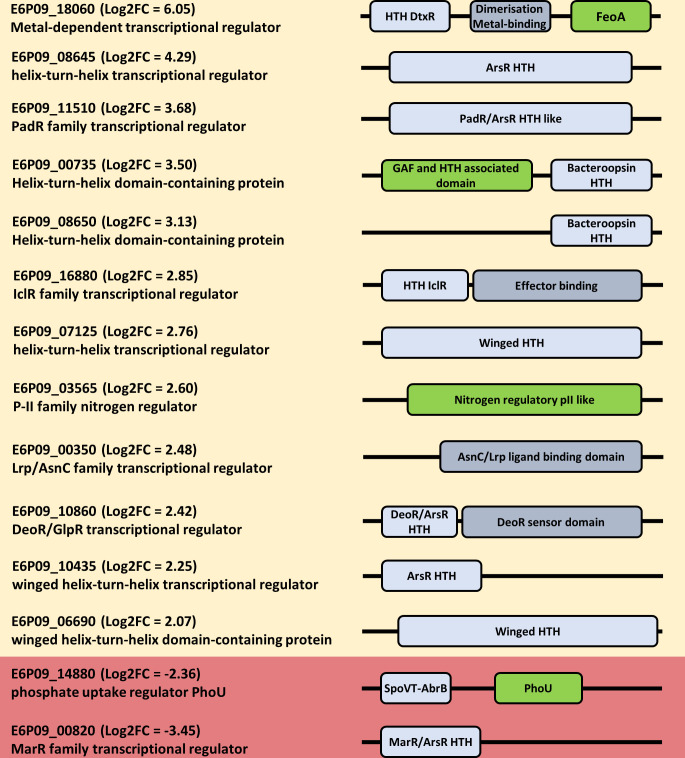
Domain scheme of transcriptional regulators with differential expression. Yellow background, regulator with log_2_FC > +2; red background, regulators with log_2_FC < −2. HTH-domains, blue; ligand binding domains, gray; other domains, green. The length and location of the domains are an approximation.

The regulatory network under denitrification has been studied deeply in several bacteria from different genera but in *H. mediterranei* is completely unknown. This analysis has delved into this, and all the identified differentially expressed transcriptional regulators were selected for further analysis of their different domains to know in which processes they might be involved (Fig. 6).

*Metal-dependent transcriptional regulator*. This regulator had the higher positive log_2_FC among all the identified regulators (Fig. 6). It belongs to the DtxR family of transcriptional repressors, that are well studied in pathogenic bacteria. These regulators were first studied in *Corynebacterium diphtheriae* because they control the expression of the *tox* operon involved in the biosynthesis of toxins (48, 49). This control is mediated by the presence of iron, which acts as a ligand molecule (48, 49). However, in other studies has been discovered that DtxR regulators not only control pathogenesis genes but also metal homeostasis in other organisms, including haloarchaea (50–52). The regulator found in this analysis possesses three domains: the HTH domain with DNA binding capacity, a ligand binding together with a dimerization domain, and a FeoA domain. These characteristics allow us to assign this regulator as a SirR transcriptional repressor. SirR usually responds to changes in iron concentration, as has been shown in other haloarchaea such as *Halobacterium salinarum*, but in this case, the oxic and denitrifying cultures had the same composition (therefore same iron concentration) (50, 52). We also observed that a set of differentially expressed genes related to iron uptake are repressed under denitrifying conditions. These genes are listed in Table 2. This is a very interesting behavior because it opens new questions about how iron homeostasis and denitrification are connected. Thus, this SirR repressor could be a possible regulator of this set of genes.

**TABLE 2:** Differentially repressed genes under denitrifying conditions related to iron uptake (oxic *vs* denitrifying conditions)

NCBI locus tag	Gene product	log_2_FC
E6P09_00145	ABC transporter substrate-binding protein (ferrichrome-binding protein)	−3.65
E6P09_02475	ABC transporter ATP-binding protein (iron/cobalamin ABC-type transporter ATP-binding protein)	−3.04
E6P09_02480	Iron ABC transporter permease (iron/cobalamin ABC transporter permease)	−4.04
E6P09_02485	NAD(P)/FAD-dependent oxidoreductase	−4.06
E6P09_11455	ABC transporter substrate-binding protein (putative iron transport protein)	−4.25
E6P09_12005	ABC-type iron (III) transport system (iron ABC transporter substrate-binding protein)	−2.12
E6P09_17310	ABC transporter substrate-binding protein (ferrichrome-binding protein)	−3.20
E6P09_17315	Iron transport protein (putative iron transport protein)	−4.11
E6P09_18530	IucA/IucC family siderophore biosynthesis protein	−6.01
E6P09_18535	Lysine N (6)-hydroxylase/L-ornithine N (5)-oxygenase family protein (siderophore biosynthesis protein)	−6.79
E6P09_18540	Acetyltransferase (siderophore biosynthesis protein)	−7.09
E6P09_18545	Iron transporter (IucA/IucC family siderophore biosynthesis protein) (siderophore biosynthesis protein)	−6.88
E6P09_18550	Aspartate aminotransferase family protein (siderophore biosynthesis protein)	−6.24
E6P09_18555	Diaminobutyrate-2-oxoglutarate transaminase (siderophore biosynthesis protein)	−6.36

*PadR transcriptional regulator–GvpE Gas vesicle regulator:* The production of gas vesicles is a strategy that allows some haloarchaea to float in the water column migrating to regions with optimal conditions (53). GvpE and GvpD are the known regulatory proteins that control the production of gas vesicles and GvpA and GvpC are the major components of them (54). In previous studies carried out in *H. mediterranei,* it has been found that the transcript levels of *gvpA* and *gvp*D genes are lower under denitrifying conditions compared to oxic conditions, and therefore the formation of gas vesicles is suppressed (55). Intriguingly, the data contrast with what has been found in the results of the present study, where the two gas vesicle gene clusters (*gvpACNO* and *gvpDEFGHIJKLM*) were differentially activated under anoxic conditions (Fig. 6 shows the log_2_FC of *gvpE* gene). In contrast with the growth conditions described in this work, Hechler and Pfeifer (55) grew *H. mediterranei* in a different media, and the anoxic conditions were set up by flushing gas into the media (55). The differences observed could be due to a more gradual transition to anoxia in the present study (and probably more similar to what happens in its natural environments). Maybe this gradual oxygen depletion could be the trigger for the gas vesicle synthesis as preparation for anoxia, being an alert signal for the cell to be adapted to a new condition in which these genes are expressed meanwhile oxygen is present. However, a fast transference to oxygen-restrictive conditions does not allow the cell to produce the whole gas vesicle machinery.

*Bacterioopsin activators*. Bacterioopsin activators are named due to their relationship to the activation of the *bop* gene cluster in *H. salinarum* (56, 57). This cluster encodes the genes of the bacterioopsin biosynthesis, the bacteriorhodopsin proton pump, and its expression is induced in low oxygen concentration and availability of light conditions by the *bat* activator (56, 58). The *bat* activator is characterized by a PAS domain, a redox sensory motif, a GAF domain, a light-responsive cyclic GMP-binding motif, an HTH domain, and an associated domain between the HTH and the GAF domain (21, 59). In *H. mediterranei,* two genes that encode for transcriptional regulators similar to bacterioopsin activators with differential expression, E6P09_08650 and E6P09_00735, have been found (Fig. 6). Intriguingly, both gene products lack the PAS and GAF domain and the E6P09_08650 also lacks the associated domain. However, other well-characterized bacterioopsin activators related to anaerobic metabolisms from different haloarchaea showed the lack of these domains too, as the NarO regulator related to the activation of denitrifying genes in *H. volcanii*, and the DmsR regulator that activates the DMSO respiration in *H. salinarum,* (21, 60). Furthermore, as the bacterioopsin regulators found in other microorganisms, the genes found in *H. mediterranei* are also classified as dimethyl sulfoxide reductase transcriptional activators by the PANTHER classification system (61). Regarding these characteristics, it can be concluded that is worth further studying these two regulators, as they may be the main regulators of the denitrification pathway in *H. mediterranei* due to their similarities with NarO and DmsR regulators. Especially, the E6P09_00735 gene due to its genomic location, since it is part of the *nirK* and *nor* genetic cluster, as it is discussed in the next section (Fig. 3; Table S2 to S4).

*Nitrogen assimilation proteins:* Some genes related to ammonium assimilation showed differential expression levels. The genes encoding the regulatory proteins GlnK1 and GlnK2, and the high-affinity ammonium transporters Amt1 and Amt2, are overexpressed in anoxic conditions (Table 3). GlnK proteins (PII proteins) are signaling proteins involved in the sensing of cellular C/N and ATP/ADP ratios and are widely distributed among *Bacteria* and *Archaea* and they have also been found in plastids of plants (62, 63). They regulate the intake of ammonium through the Amt transporter as well as the glutamine synthetase activity (64). In *H. mediterranei*, *glnk1* is co-transcribed with *amt1* and *glnk2* with *amt2*, and their transcription is enhanced in the absence of ammonium (65). The increase in the expression of these genes in the present study could be due to the shift to anoxic conditions or the decrease in ammonium levels during the growth, although under the anoxic conditions, ammonium was still the nitrogen source, as no increase in the transcription of nitrate assimilation genes was detected. The decrease in ammonium concentration may also explain the increase in the transcription of the gene encoding glutamate synthase, *gltS*. The glutamine synthetase (GlnA)–glutamate synthase (GltS) pathway is preferential for ammonium assimilation when ammonium concentration is low, while in ammonium abundance, assimilation occurs preferentially *via* glutamate dehydrogenase. However, there is no increase in *glnA* transcription and no decrease in glutamate dehydrogenase, *gdh1,* transcription (Table 3), which is highly repressed under ammonium starvation (65). Probably, these changes in gene expression occur at lower ammonium concentrations than those obtained in the present study, but what is most noteworthy is the high transcriptional repression of *glnA2* and *glnA3* genes. These genes show a high homology with glutamine synthetase (GlnA), but it has been proposed that their role was different (66). Although the role of GlnA3 has not yet been described, GlnA2 is known to have glutamate–putrescine ligase activity and could be responsible for the growth of *H. mediterranei* in the presence of putrescine as the sole source of nitrogen and carbon (67). The transcriptional repression of both genes does not appear to be related to the decrease in ammonium concentration, as previous analyses of *H. mediterranei*’s response to ammonium starvation did not reveal a reduction in the transcription of *glnaA*2 or *glnA*3. Therefore, the role of these two genes in the response to anoxia is unclear but could be due to a possible role of polyamines, such as putrescine, as metabolic regulators. It is known that polyamines stimulate the synthesis of some proteins and increase the fidelity of translation (68). In *Escherichia coli*, the effect of putrescine in gene transcription was studied, showing that some genes up-regulated were involved in iron uptake and energy metabolism, whereas down-regulated genes were related to amino acid metabolism or biosynthesis of cofactors and carriers (68). Similar effects could be given in *H. mediterranei*.

**TABLE 3:** Expression changes of the genes related to nitrogen assimilation in *H. mediterranei* under denitrifying conditions (oxic *vs* denitrifying conditions)

NCBI locus tag	Gene product	log_2_FC
E6P09_01670	Ferredoxin-nitrite reductase (NasD)	0.59
E6P09_01675	Molybdenum cofactor guanylyltransferase (NasC)	0.84
E6P09_01680	MFS (nitrate) transporter (NasB)	0.01
E6P09_01685	Assimilative nitrate reductase (NasA)	−0.89
E6P09_04325	GlnA	0.37
E6P09_11460	GlnA2 (glutamate–putrescine ligase)	−4.05
E6P09_11450	GlnA3	−5.37
E6P09_07245	Glutamate synthase large subunit (GltS)	2.38
E6P09_03560	Ammonium transporter (AmtB2)/ P-II family nitrogen regulator (GlnK2)^*a*^	1.98
E6P09_03565	P-II family nitrogen regulator (GlnK1)	2.60
E6P09_03570	Ammonium transporter (AmtB1; annotated as pseudogene)	2.13
E6P09_00800	Glu/Leu/Phe/Val dehydrogenase (GdhA1)	−0.45

^*a*^ *amtB*2 and *glnK*2 genes are misannotated as a single transcript for *amtB*2.

*ArsR transcriptional regulators:* The ArsR family of transcriptional regulators is widely distributed not only in *Bacteria* but also in the *Archaea* domain, representing the third more abundant family of transcriptional regulators (69). Despite its abundance and contrary to the bacterial counterpart, the ArsR family in *Archaea* is not well studied and the information about it is limited. Mainly, these regulators are involved in heavy metal stress response, but in *Archaea,* they have also acquired other functions (70, 71). However, up to date, there is no direct relationship between these regulators and the nitrogen cycle. In gram-positive bacteria, an ArsR repressor called SufR has been related to iron-sulfur cluster assembly but not in *Archaea* (72). The results of this study show that there were five differentially expressed regulators from this family, suggesting that some of these regulators are directly or indirectly involved in the anoxic/denitrifying response (Fig. 6). Therefore, more studies are needed to understand the specific role of these regulators and discover which genes are under their regulation.

*Other transcriptional regulators:* In this study, other regulators from different families have shown differential expression in denitrifying conditions concerning oxic conditions. These are AsnC/Lrp, IclR, and PhoU regulators. AsnC/Lrp regulators are the most abundant in *Archaea*, they are considered global regulators that can act as activators or repressors and are involved in the regulation of many functions (69, 73). They are well studied, and even in *H. mediterranei* there are studies of specific AsnC/Lrp regulators which has been linked with the assimilation of nitrogen, but no relationship with denitrification has been established (74, 75). IclR regulators are also involved in different processes and present a dual function acting not only as activators but also as repressors. As the AsnC/Lrp regulators, they are involved in different processes, such as carbon metabolisms, amino acid, and antibiotic production or quorum sensing, but no direct relationship with denitrification has been found (76–79). Finally, PhoU is a regulator related to phosphate uptake, it has been studied in bacteria and the haloarchaeon *H. salinarum,* showing an increase in its expression (together with other phosphate metabolisms related genes called the PHO stimulon) when cells are grown under phosphate starvation (80, 81). This is not the case in our study because phosphate concentration in the media is the same in all conditions. However, we have not measured the intracellular concentration and maybe during anoxic/denitrifying conditions could suffer some changes derived from the repression of this gene.

### Genes encoding accessory proteins could share their transcriptomic regulation with the main denitrification genes

Previous studies found a regulatory motif (CGAAYATDKTYG) in the promoter regions of the main denitrification genes and other genes in the *H. mediterranei* genome, using the bioinformatic tool FIMO (21, 23, 82). These other genes include previously identified accessory proteins and a number of other proteins that have not been formally linked to the denitrification process (20). The list of the promoter regions with the consensus sequence was used to look for regions under its regulation that were differentially expressed under denitrifying conditions (log_2_FC < −2 or log_2_FC > +2 and padj <0.05). The screening showed that 25 promoter regions displayed differentially expressed genes under its control (Table S5).

As expected, among these differentially expressed genes are the ones encoding the main enzymes (*nar* operon, *nirK, nor,* and *nos* operon). However, other genes in this list encode proteins that have been mentioned and discussed for the first time in this article as possible accessory proteins to denitrification in haloarchaea. These genes are the bacterioopsin activator gene E6P09_00735, the cyanoglobin gene E6P09_11805 and two out of four genes encoding DUF2249 domain containing-proteins (E6P09_00770 and E6P09_17415 genes). This finding strengthens the hypothesis that these proteins are involved in denitrification metabolism. Intriguingly, this list also contains two genomic organizations in which the motif was repeated in different promoter regions and showed a high number of genes with a positive log_2_FC value. The first organization is located on the main chromosome is about 12.5 kb (coordinates 142,837 to 155,489) and covers the region between E6P09_00735 and E6P09_00790 genes. This region has eight repeats of the motif in different positions (Fig. 3; Table S2 to S4). In addition, this gene cluster includes not only the *nirK* gene and *nor* gene, but also two genes encoding for proteins containing the DUF2249 domain (genes E6P09_00770 and E6P09_00795), the bacterioopsin activator E6P09_00735 and halocyanins that can act as electron donors (E6P09_00740 and E6P09_00760). The second organization is located on the pHME322 megaplasmid, is about 26.8 kb, and is found between the E6P09_17385 gene and the beginning of the megaplasmid (coordinates 1 to 2,079) and E6P09_17435 gene and the end of the megaplasmid. This region has five repetitions of the motif, and it includes the *nar* and *nos* operons and the other two genes encoding DUF2249 domain-containing proteins (E6P09_17415 and E6P09_17395 genes) (Fig. 3; Table S2 to S4).

Most of the genes present in these two regions showed positive differential expression under denitrification conditions and their genomic location links different proteins that have been proposed to have a role in denitrification, such as the DUF2249 domain-containing proteins and the bacterioopsin activator (E6P09_00735). Although an evolutionary analysis is outside the scope of this article, these two delimited regions can give more information about the evolution of denitrification in haloarchaea if they are analyzed in depth. However, it is worth noting that *nir* and *nor* genes have been linked in the past (9, 83). The presence of “genomic denitrification islands” in different prokaryotic genomes has been hypothesized to explain the clustering of denitrification genes and it could be the explanation of why these genes are clustered in *H. mediterranei* (83). In addition, the analysis of these clusters can be used to identify new genes related to the denitrification pathway, as is the case in this research, exploiting the idea that gene clustering is due to the fact that they code for proteins that interact physically or functionally (83–85).

### Metal homeostasis is widely controlled under denitrifying conditions

Metals such as copper, iron, manganese, molybdenum, and nickel are micronutrients that act as redox centers or serve as a cofactor of different proteins in the cells (86–89). However, other metals such as arsenic have no biological roles and are potentially toxic to cells (90). Consequently, metal homeostasis is an important process for maintaining cell integrity, and haloarchaea have developed different strategies for dealing with metallic compounds (87, 88).

Expression analysis of different gene clusters under denitrifying conditions denoted that there is strict control of metal homeostasis in *H. mediterranei* cells. In the previous section, it was reported that a regulator of the DtxR family of transcriptional repressors showed a high log_2_FC value under this condition, and it may be involved in the repression of the iron uptake of the cell. However, iron is not the only metal that seems to be regulated in this study. One of the two Zn uptake systems (ZnuABC) encoded in the *H. mediterranei* genome showed a highly positive expression change (Table 4). The Zn uptake system in haloarchaea is similar to the bacterial system. It is accomplished by a membrane permease (ZnuB), an ATPase (ZnuC), and a soluble periplasmic protein that captures Zn (II) and moves it to the membrane component ZnuB (91). This system in haloarchaea has been studied in the microorganisms *H. volcanii*, and it has been proposed that it is regulated by an sRNA (sRNA479) that negatively regulates its expression (92). sRNA479 does not have any homologous in *H. mediterranei*, but there are other sRNAs identified in the *H. mediterranei* genome that are in the same genetic position as sRNA479 (immediately downstream a CAS gene cassette). These are sRNA112, sRNA113, and sRNA114 (93). Other interesting gene clusters related to metals that showed highly positive differential expression are two operons that encode an electron donor (plastocyanin or halocyanin), a transmembrane protein of unknown function that showed similarity to metal transporters (data not shown) and a multicopper oxidase domain-containing protein (Table 4). These gene clusters probably are related to copper homeostasis, but multicopper oxidases have shown different roles and more experimental procedures would be needed to understand their role in this study (94). Another remarkable gene cluster with differential expression is encoding an SCO family protein together with a hypothetical protein, a thioredoxin family protein, and a cytochrome c biogenesis protein (Table 4). On the one hand, SCO proteins are involved in copper homeostasis, delivering Cu to other enzymes, or participating in protection against oxidative stress (95). On the other hand, it is known that NosZ possesses copper centers in its structure, and these centers need a maturation process to be active (96). One of the steps of this maturation process is the reduction of disulfide bonds present in the chaperone of the CuA center of NosZ and this reduction is carried out by an SCO protein (97, 98). There is no evidence that this SCO protein is the protein in charge of this maturation of NosZ in *H. mediterranei*, but its gene is the only one encoding an SCO protein with high expression under denitrifying conditions. Furthermore, it is worth mentioning the *acnA* gene (E6P09_05600) encoding the aconitase enzyme from the tricarboxylic acid cycle is the only protein from this pathway that was differentially activated (Log_2_FC = 2.89) under denitrifying conditions (Table S6). In previous studies from mammals and some bacteria, it has been reported that this protein loses its aconitase activity by losing its [4Fe–4S] center under oxidative stress or iron deprivation (99). This event acts as a switch, enabling aconitase to bind RNA and regulate genes related to the iron response (100). This behavior has not been described in *Archaea* but looking at the changes in different iron genes during denitrification it would be interesting to explore the mRNA binding capacity of the archaeal aconitase to deep more in the metal homeostasis of this kind of microorganisms. Finally, an operon encoding molybdenum-related genes also showed upregulation under denitrifying conditions. This operon includes the molybdate transporter *modA* gene (E6P09_00610) and the *mobA* gene (E6P09_00615) involved in the biosynthesis of the molybdenum cofactor of some enzymes such as the respiratory nitrate reductase (89, 101).

**TABLE 4:** Expression changes of different metal-related genes and their gene clusters under denitrifying conditions (oxic *vs* denitrifying conditions)

	NCBI locus tag	Gene product	Log_2_FC
Operon	E6P09_14780	Metal ABC transporter permease (ZnuB)	4.63
	E6P09_14785	Metal ABC transporter ATP-binding protein (ZnuC)	4.35
	E6P09_14790	Metal ABC transporter substrate-binding protein (ZnuA)	4.34
Operon	E6P09_18045	Multicopper oxidase domain-containing protein	7.66
	E6P09_18050	Hypothetical protein (transmembrane protein)	7.29
	E6P09_18055	Copper-binding plastocyanin-like protein	7.10
Operon	E6P09_05590	Hypothetical protein (halocyanin)	2.80
	E6P09_05585	Hypothetical protein (transmembrane protein)	2.60
	E6P09_05580	Multicopper oxidase domain-containing protein	3.23
Operon	E6P09_05575	Hypothetical protein (cupredoxin domain-containing protein)	7.35
	E6P09_05570	SCO family protein	7.56
	E6P09_05565	Thioredoxin family protein	6.51
	E6P09_05560	Cytochrome c biogenesis protein	3.05
Operon	E6P09_00620	Molybdopterin-dependent oxidoreductase	3.57
	E6P09_00615	Molybdenum cofactor guanylyltransferase (MobA)	2.37
	E6P09_00610	Substrate-binding domain-containing protein (ModA)	2.54
	E6P09_00605	ABC transporter permease	3.61

In summary, metal homeostasis under denitrifying conditions is highly regulated in haloarchaea, especially the metabolism of iron, copper, molybdenum, and zinc showed differences in genes related to their metabolisms. This control is probably because cells need a balance between the requirement of cofactors to build all the key enzymes as well as accessory proteins necessary to perform denitrification (many of their structures involved MoCo cofactors, Fe-S clusters, and/or Cu centers) and the redox stress that a high concentration of metals could produce.

In conclusion, the transcriptomic analysis conducted in this study has provided comprehensive insights into the metabolic state of the microorganism under denitrifying conditions. The observed reduction in metabolic activity can be attributed to the inhibition of crucial pathways such as replication, transcription, and translation. Moreover, our investigation has established a link between denitrification and the regulation of metal homeostasis, as evidenced by substantial alterations in the expression of genes associated with metal-related processes. This phenomenon can be attributed to the fact that denitrification enzymes are metalloproteins, requiring optimal intracellular metal concentrations and redox balance for efficient functionality.

**Fig 7:**
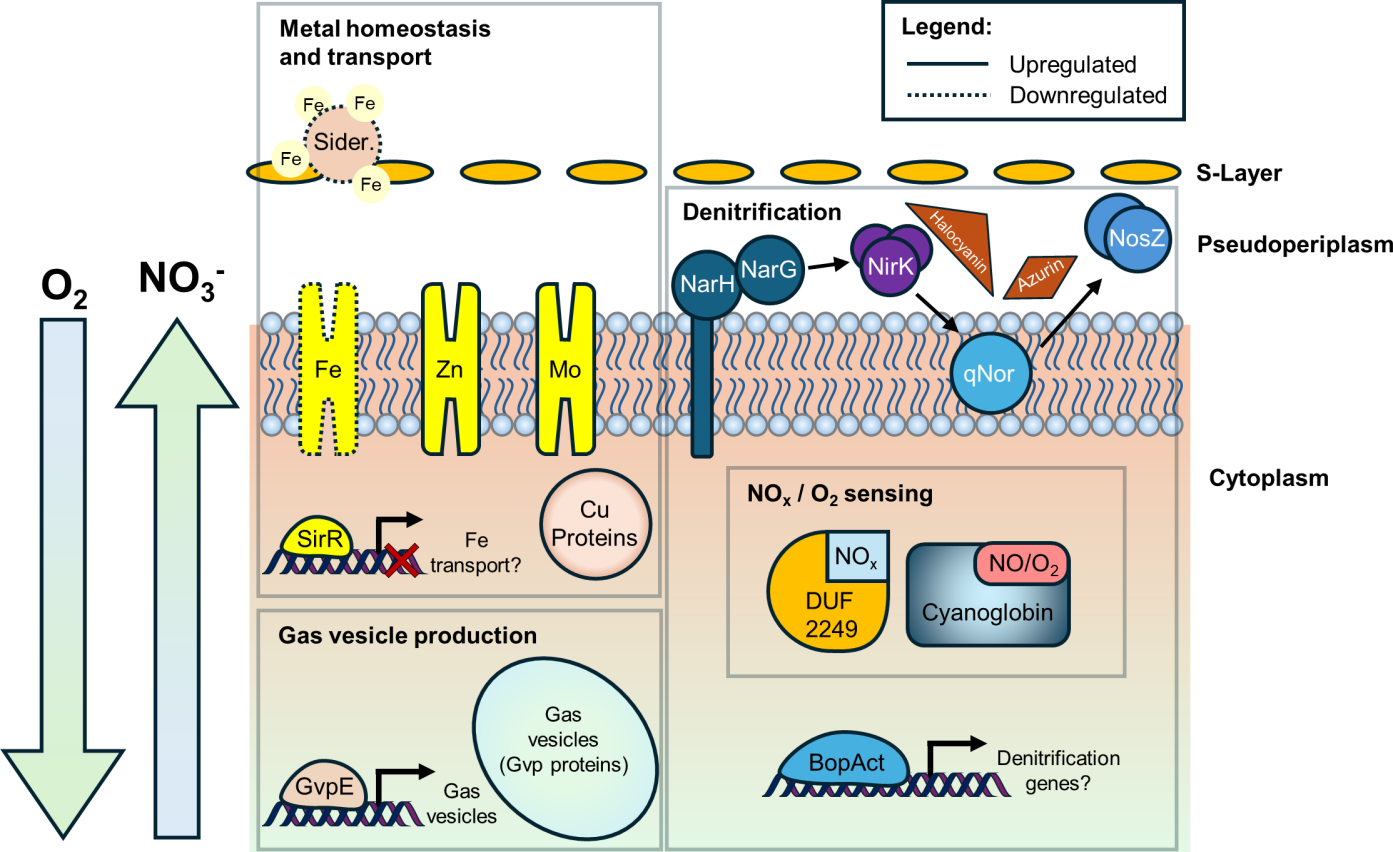
Schematic representation of the regulation and the adaptation mechanisms identified by RNA-Seq analysis of *H. mediterranei* cells under denitrifying conditions. Gene products encoded by differentially upregulated (continuous figure shape) and downregulated genes (discontinuous figure shape) are represented. SirR and BopAct are transcriptional regulators with an assigned putative function. Abbreviations: Sider., siderophores; BopAct., bacterioopsin activator; DUF2249, Domain of Unknown Function 2249-containing protein.

Furthermore, our analyses have unveiled several genes encoding proteins implicated in denitrification processes. Notably, bacterioopsin activators have emerged as potential key regulators of essential denitrification genes, while proteins harboring the DUF2249 domain and cyanoglobin appear to serve as putative sensors for denitrification-related nitrogen species, with the latter possibly functioning as an oxygen sensor as well. An additional intriguing aspect addressed in this study is the presence of regulatory motifs associated with denitrification in activated genes, reinforcing the notion of a coordinated and shared regulatory network governing these genes under denitrifying conditions. Moreover, up-regulation of genes encoding proteins related to gas vesicle production was observed, suggesting that cells employ this strategy to reach zones of higher oxygen in the water column. All these adaptations are summarized in Fig. 7.

The primary objective of this analysis is to serve as a valuable resource for future molecular studies, facilitating a deeper understanding of the genetic regulation and metabolic adaptations exhibited by haloarchaea in response to denitrification. The identification of potential targets for future investigations is explicitly delineated in this work, offering a roadmap for advancing our knowledge in this area of microbial physiology.

## MATERIALS AND METHODS

### Cell growth, RNA extraction, and rRNA depletion

*H. mediterranei* strain R4 was grown in defined media supplemented with nitrate: 27.75 mM (0.5% wt/vol) glucose, 1 mM Pi (K_2_HPO4/KH_2_PO4), 15 mM NH_4_Cl, 10 mM KNO_3_, 0.03 mM FeCl_3_, 2.67 M NaCl, 0.16 M MgSO_4_·7H_2_O, 0.133 M MgCl_2_·6H_2_O, 53.33 mM KCl, 1.6 mM NaHCO_3_, 4.53 mM NaBr, 6.6 mM CaCl_2_, and buffered with 50 mM MOPS ((3-(N-morpholino) propanesulfonic acid). Cultures were set up in two different ways, aerobiosis and denitrifying conditions, both at pH 7.3 (adjusted with NaOH) and 42°C.

Aerobic cultures were grown in an Erlenmeyer flask, with shaking (170 rpm) and with a high air chamber (90% of flask volume) to ensure high oxygen exchange with the media. These cultures were grown to an optical density at 600 nm (OD_600 nm_) around 0.2 and then moved to denitrifying conditions. For setting up this condition, the cultures were transferred for 36 hours to closed flasks. These flasks were filled completely without leaving an air chamber in the head space to not allow gas exchange. All the cultures were conducted in triplicate.

RNA extraction was performed using the QIAGEN RNeasy Kit. Samples were taken from three biological replicates in both conditions: just before the culture switches to the denitrifying condition (OD_600 nm_ = 0.20–0.24), and after 36 hours of growing under this condition (OD_600 nm_ = 0.64–0.70). RNA from each sample was treated with Invitrogen Kit TURBO DNA-free. 11 µg of RNA sample was digested in a 30 µL reaction using 8 Units (U) of TURBO DNAse. First, the samples were incubated at 37°C for 1 hour with 4 U of TURBO DNAse, then four more units were added, and the sample was incubated for one additional hour. DNA digestion result was checked by 2% TAE agarose gel and by PCR using primer pairs for three different genomic regions of the *H. mediterranei* genome (regions were selected randomly; Table S7). Moreover, all samples were analyzed before and after DNAse treatment by Agilent Bioanalyzer with an RNA 6000 Pico Kit, High Sensitivity RNA ScreenTape assay (RIN for all samples before DNAse treatment was >9 to be validated).

Finally, rRNA from samples was depleted using riboPOOL-specific probes for *H. volcanii* (siTOOLS Biotech) following the manufacturer’s instructions. 10 ng of RNA from each sample was used for the depletion. rRNA depletion was checked by Agilent Bioanalyzer with an RNA 6000 Pico Kit, High Sensitivity RNA ScreenTape assay.

### RNA-Seq protocol, data processing, mRNA differential expression, and gene enrichment analysis

Depleted samples were sent to Microomics Systems S.L (Barcelona, Spain) for library construction using NEBNext® Ultra II Directional RNA Library Prep Kit for Illumina, and sequencing using Illumina Hiseq 2500 2 × 125.

Sequencing data were quality-checked using FastQC v0.11.8 and adaptors were trimmed by Trimmomatic v0.39 (102, 103). Pre-processed data were aligned to the reference genome using Bowtie2 v2.3.5.1 and SAM files were converted to BAM files and indexed using SAMtools (104). The quality of the alignment was checked by qualimap v.2.2.2, and the count table was built with featureCounts v1.6.4 (105, 106). Differential expression analysis between aerobic and denitrifying conditions was performed using DESeq2 v1.24.0 and Gene Set Enrichment Analysis (GSEA) was used to detect which KEGG pathways were enriched in the gene list derived from DESeq2 analysis (26, 107, 108). R package clusterProfiler v3.12.0 was used for this purpose using a gene expression filter of padj <0.05 (109). The expression changes of the genes encoding the main denitrification genes (*narG, nirK, nor,* and *nosZ*) were used as direct markers for the validation of the study. Finally, RNA-Seq data visualization was done using Integrative Genomics Viewer (IGV) software v2.13 (110).

### Denitrification-related genes search by FIMO

Denitrification-related genes found in the genome of *H. mediterranei* in a previous study were compared with the transcriptomic analyses of the current study (23). From that study, a set of 82 promoter regions that shared the regulatory motif CGAAYATDKTYG was used for further analyses. This motif had been found in the promoter regions of the *nar* operon, *nirK* gene, *nor* gene, and *nos* operon in the mentioned study. Subsequently, the genes under the regulation of these promoter regions were used to identify the log_2_FC (FC: Fold Change) of its expression under denitrifying conditions. Finally, the genes that showed differential expression were filtered for further discussion.

### Sequence alignments

Protein sequences of the gene products of the DUF2249 containing proteins present in the *H. mediterranei* genome and DrpA of *T. thermophilus* were downloaded from the UniProt Database (111). Sequences were aligned (default parameters were used) and afterward, a phylogenetic neighbor-joining tree (Bootstrap 500) was built with these alignments using MEGA 11 software (112).

## Data Availability

The RNA-seq data generated from this study are deposited in NCBI’s Gene Expression Omnibus (GEO) and are available under the accession number GSE245042.

## SUPPLEMENTAL MATERIAL

Supplemental material aem.00571-24-s0001.docx Tables S1 to S7.

ASM does not own the copyrights to Supplemental Material that may be linked to, or accessed through, an article. The authors have granted ASM a non-exclusive, world-wide license to publish the Supplemental Material files. Please contact the corresponding author directly for reuse.

## References

[1] Torregrosa-Crespo J, Bergaust L, Pire C, Martínez-Espinosa RM. 2018. Denitrifying haloarchaea: sources and sinks of nitrogenous gases. FEMS Microbiol Lett 365. doi:10.1093/femsle/fnx270 29237000

[2] Schuler MS, Cañedo-Argüelles M, Hintz WD, Dyack B, Birk S, Relyea RA. 2018. Regulations are needed to protect freshwater ecosystems from salinization. Philos Trans R Soc Lond B Biol Sci 374:20180019. doi:10.1098/rstb.2018.0019 30509918 PMC6283961

[3] Delgado-Baquerizo M, Maestre FT, Gallardo A, Bowker MA, Wallenstein MD, Quero JL, Ochoa V, Gozalo B, García-Gómez M, Soliveres S, et al.. 2013. Decoupling of soil nutrient cycles as a function of aridity in global drylands. Nature 502:672–676. doi:10.1038/nature12670 24172979

[4] Singh A, Singh AK. 2017. Haloarchaea: worth exploring for their biotechnological potential. Biotechnol Lett 39:1793–1800. doi:10.1007/s10529-017-2434-y 28900776

[5] Martínez GM, Pire C, Martínez-Espinosa RM. 2022. Hypersaline environments as natural sources of microbes with potential applications in biotechnology: the case of solar evaporation systems to produce salt in Alicante County (Spain). Curr Res Microb Sci 3:100136. doi:10.1016/j.crmicr.2022.100136 35909606 PMC9325878

[6] Ventosa A, de la Haba RR, Sánchez-Porro C, Papke RT. 2015. Microbial diversity of hypersaline environments: a metagenomic approach. Curr Opin Microbiol 25:80–87. doi:10.1016/j.mib.2015.05.002 26056770

[7] Kasirajan L, Maupin-Furlow JA. 2021. Halophilic archaea and their potential to generate renewable fuels and chemicals. Biotechnol Bioeng 118:1066–1090. doi:10.1002/bit.27639 33241850 PMC7897274

[8] Ventosa A, Fernández AB, León MJ, Sánchez-Porro C, Rodriguez-Valera F. 2014. The Santa Pola saltern as a model for studying the microbiota of hypersaline environments. Extremophiles 18:811–824. doi:10.1007/s00792-014-0681-6 25129545

[9] Miralles-Robledillo JM, Bernabeu E, Giani M, Martínez-Serna E, Martínez-Espinosa RM, Pire C. 2021. Distribution of denitrification among haloarchaea: a comprehensive study. Microorganisms 9:1–14. doi:10.3390/microorganisms9081669 PMC8400030 34442748

[10] Ehhalt D, Prather M, Dentener F, Derwent R, Dlugokencky E, Holland E, Isaksen I, Katima J, Kirchhoff V, Matson P, et al.. 2001. Atmospheric chemistry and greenhouse gases, p 241–287. In Houghton JT, Ding Y, Griggs DJ, Noguer M, Dai X, Maskell K, Johnson CA (ed), Climate change 2001: the scientific basis. contribution of working group I to the third assessment report of the intergovernmental panel on climate change. Cambridge University Press, Cambridge, New York.

[11] Edbeib MF, Wahab RA, Huyop F. 2016. Halophiles: biology, adaptation, and their role in decontamination of hypersaline environments. World J Microbiol Biotechnol 32:135. doi:10.1007/s11274-016-2081-9 27344438

[12] Andrei AŞ, Banciu HL, Oren A. 2012. Living with salt: metabolic and phylogenetic diversity of archaea inhabiting saline ecosystems. FEMS Microbiol Lett 330:1–9. doi:10.1111/j.1574-6968.2012.02526.x 22339687

[13] Torregrosa-Crespo J, Pire C, Martínez-Espinosa RM, Bergaust L. 2019. Denitrifying haloarchaea within the genus Haloferax display divergent respiratory phenotypes, with implications for their release of nitrogenous gases. Environ Microbiol 21:427–436. doi:10.1111/1462-2920.14474 30421557

[14] Lledó B, Martínez-Espinosa RM, Marhuenda-Egea FC, Bonete MJ. 2004. Respiratory nitrate reductase from haloarchaeon Haloferax mediterranei: biochemical and genetic analysis. Biochim Biophys Acta 1674:50–59. doi:10.1016/j.bbagen.2004.05.007 15342113

[15] Martinez-Espinosa RM, Bonete MJ. 2007. Bioremediation of chlorate and perchlorate salted water using Haloferax mediterranei. J Biotechnol 131:S227. doi:10.1016/j.jbiotec.2007.07.411

[16] Martinez-Espinosa RM, Dridge EJ, Bonete MJ, Butt JN, Butler CS, Sargent F, Richardson DJ. 2007. Look on the positive side! The orientation, identification and bioenergetics of “Archaeal” membrane-bound nitrate reductases. FEMS Microbiol Lett 276:129–139. doi:10.1111/j.1574-6968.2007.00887.x 17888006

[17] Nájera-Fernández C, Zafrilla B, Bonete MJ, Martínez-Espinosa RM. 2012. Role of the denitrifying haloarchaea in the treatment of nitrite-brines. Int Microbiol 15:111–119. doi:10.2436/20.1501.01.164 23847815

[18] Esclapez J, Zafrilla B, Martínez-Espinosa RM, Bonete MJ. 2013. Cu-NirK from Haloferax mediterranei as an example of metalloprotein maturation and exportation via Tat system. Biochim Biophys Acta 1834:1003–1009. doi:10.1016/j.bbapap.2013.03.002 23499847

[19] Torregrosa-Crespo J, Pire C, Bergaust L, Martínez-Espinosa RM. 2020. Haloferax mediterranei, an archaeal model for denitrification in saline systems, characterized through integrated physiological and transcriptional analyses. Front Microbiol 11:768. doi:10.3389/fmicb.2020.00768 32390995 PMC7188791

[20] Torregrosa-Crespo J, Pire C, Richardson DJ, Martínez-Espinosa RM. 2020. Exploring the molecular machinery of denitrification in Haloferax mediterranei through proteomics. Front Microbiol 11:605859. doi:10.3389/fmicb.2020.605859 33363526 PMC7754194

[21] Hattori T, Shiba H, Ashiki K, Araki T, Nagashima Y, Yoshimatsu K, Fujiwara T. 2016. Anaerobic growth of haloarchaeon Haloferax volcanii by denitrification is controlled by the transcription regulator NarO. J Bacteriol 198:1077–1086. doi:10.1128/JB.00833-15 26787768 PMC4800875

[22] Tavares P, Pereira AS, Moura JJG, Moura I. 2006. Metalloenzymes of the denitrification pathway. J Inorg Biochem 100:2087–2100. doi:10.1016/j.jinorgbio.2006.09.003 17070915

[23] Miralles-Robledillo JM, Martínez-Espinosa RM, Pire C. 2023. Analysis of the external signals driving the transcriptional regulation of the main genes involved in denitrification in Haloferax mediterranei. Front Microbiol 14:1109550. doi:10.3389/fmicb.2023.1109550 37007523 PMC10062603

[24] Carbon S, Douglass E, Good BM, Unni DR, Harris NL, Mungall CJ, Basu S, Chisholm RL, Dodson RJ, Hartline E. 2021. The Gene Ontology resource: enriching a GOld mine. Nucleic Acids Res 49:D325–D334. doi:10.1093/nar/gkaa1113 33290552 PMC7779012

[25] Ashburner M, Ball CA, Blake JA, Botstein D, Butler H, Cherry JM, Davis AP, Dolinski K, Dwight SS, Eppig JT, Harris MA, Hill DP, Issel-Tarver L, Kasarskis A, Lewis S, Matese JC, Richardson JE, Ringwald M, Rubin GM, Sherlock G. 2000. Gene ontology: tool for the unification of biology. The Gene Ontology Consortium. Nat Genet 25:25–29. doi:10.1038/75556 10802651 PMC3037419

[26] Kanehisa M, Goto S, Sato Y, Furumichi M, Tanabe M. 2012. KEGG for integration and interpretation of large-scale molecular data sets. Nucleic Acids Res 40:D109–D114. doi:10.1093/nar/gkr988 22080510 PMC3245020

[27] Richardson D, Felgate H, Watmough N, Thomson A, Baggs E. 2009. Mitigating release of the potent greenhouse gas N_2_O from the nitrogen cycle - could enzymic regulation hold the key?. Trends Biotechnol 27:388–397. doi:10.1016/j.tibtech.2009.03.009 19497629

[28] Liu G, Cai S, Hou J, Zhao D, Han J, Zhou J, Xiang H. 2016. Enoyl-CoA hydratase mediates polyhydroxyalkanoate mobilization in Haloferax mediterranei. Sci Rep 6:24015. doi:10.1038/srep24015 27052994 PMC4823750

[29] Liu G, Hou J, Cai S, Zhao D, Cai L, Han J, Zhou J, Xiang H. 2015. A patatin-like protein associated with the polyhydroxyalkanoate (PHA) granules of Haloferax mediterranei acts as an efficient depolymerase in the degradation of native PHA. Appl Environ Microbiol 81:3029–3038. doi:10.1128/AEM.04269-14 25710370 PMC4393451

[30] Galinier A, Deutscher J. 2017. Sophisticated regulation of transcriptional factors by the bacterial phosphoenolpyruvate: sugar phosphotransferase system. J Mol Biol 429:773–789. doi:10.1016/j.jmb.2017.02.006 28202392

[31] Härtig E, Schiek U, Vollack KU, Zumft WG. 1999. Nitrate and nitrite control of respiratory nitrate reduction in denitrifying Pseudomonas stutzeri by a two-component regulatory system homologous to NarXL of Escherichia coli. J Bacteriol 181:3658–3665. doi:10.1128/JB.181.12.3658-3665.1999 10368138 PMC93841

[32] Nohno T, Noji S, Taniguchi S, Saito T. 1989. The narX and narL genes encoding the nitrate-sensing regulators of Escherichia coli are homologous to a family of prokaryotic two-component regulatory genes. Nucleic Acids Res 17:2947–2957. doi:10.1093/nar/17.8.2947 2657652 PMC317704

[33] Toyofuku M, Nomura N, Fujii T, Takaya N, Maseda H, Sawada I, Nakajima T, Uchiyama H. 2007. Quorum sensing regulates denitrification in Pseudomonas aeruginosa PAO1. J Bacteriol 189:4969–4972. doi:10.1128/JB.00289-07 17449629 PMC1913425

[34] Wang N, Gao J, Liu Y, Wang Q, Zhuang X, Zhuang G. 2021. Realizing the role of N-acyl-homoserine lactone-mediated quorum sensing in nitrification and denitrification: a review. Chemosphere 274:129970. doi:10.1016/j.chemosphere.2021.129970 33979914

[35] Bernabeu E, Miralles-Robledillo JM, Giani M, Valdés E, Martínez-Espinosa RM, Pire C. 2021. In silico analysis of the enzymes involved in haloarchaeal denitrification. Biomolecules 11:1043. doi:10.3390/biom11071043 34356667 PMC8301774

[36] Pei J, Li W, Kinch LN, Grishin NV. 2014. Conserved evolutionary units in the heme-copper oxidase superfamily revealed by novel homologous protein families. Protein Sci 23:1220–1234. doi:10.1002/pro.2503 24931479 PMC4243994

[37] Alvarez-Carreño C, Alva V, Becerra A, Lazcano A. 2018. Structure, function and evolution of the hemerythrin-like domain superfamily. Protein Sci 27:848–860. doi:10.1002/pro.3374 29330894 PMC5866928

[38] Chahlafi Z, Alvarez L, Cava F, Berenguer J. 2018. The role of conserved proteins DrpA and DrpB in nitrate respiration of Thermus thermophilus. Environ Microbiol 20:3851–3861. doi:10.1111/1462-2920.14400 30187633 PMC6282519

[39] Zumft WG. 1997. Cell biology and molecular basis of denitrification. Microbiol Mol Biol Rev 61:533–616. doi:10.1128/mmbr.61.4.533-616.1997 9409151 PMC232623

[40] Storbeck S, Rolfes S, Raux-Deery E, Warren MJ, Jahn D, Layer G. 2010. A novel pathway for the biosynthesis of heme in Archaea: genome-based bioinformatic predictions and experimental evidence. Archaea 2010:175050. doi:10.1155/2010/175050 21197080 PMC3004389

[41] Freitas TAK, Hou S, Dioum EM, Saito JA, Newhouse J, Gonzalez G, Gilles-Gonzalez MA, Alam M. 2004. Ancestral hemoglobins in Archaea. Proc Natl Acad Sci U S A 101:6675–6680. doi:10.1073/pnas.0308657101 15096613 PMC404104

[42] Lecomte JTJ, Vuletich DA, Lesk AM. 2005. Structural divergence and distant relationships in proteins: evolution of the globins. Curr Opin Struct Biol 15:290–301. doi:10.1016/j.sbi.2005.05.008 15922591

[43] Vinogradov SN, Tinajero-Trejo M, Poole RK, Hoogewijs DK. 2013. Bacterial and archaeal globins - a revised perspective. Biochim Biophys Acta 1834:1789–1800. doi:10.1016/j.bbapap.2013.03.021 23541529

[44] Thorsteinsson MV, Bevan DR, Potts M, Dou Y, Eich RF, Hargrove MS, Gibson QH, Olson JS. 1999. A cyanobacterial hemoglobin with unusual ligand binding kinetics and stability properties. Biochemistry 38:2117–2126. doi:10.1021/bi9819172 10026295

[45] Milani M, Pesce A, Ouellet H, Guertin M, Bolognesi M. 2003. Truncated hemoglobins and nitric oxide action. IUBMB Life 55:623–627. doi:10.1080/15216540310001628708 14711009

[46] Gardner PR. 2012. Hemoglobin: a nitric-oxide dioxygenase. Scientifica (Cairo) 2012:683729. doi:10.6064/2012/683729 24278729 PMC3820574

[47] Saito JA, Wan X, Lee KS, Hou S, Alam M. 2008. Globin-coupled sensors and protoglobins share a common signaling mechanism. FEBS Lett 582:1840–1846. doi:10.1016/j.febslet.2008.05.004 18486614

[48] Tao X, Schiering N, Zeng HY, Ringe D, Murphy JR. 1994. Iron, DtxR, and the regulation of diphtheria toxin expression. Mol Microbiol 14:191–197. doi:10.1111/j.1365-2958.1994.tb01280.x 7830565

[49] Parveen S, Bishai WR, Murphy JR. 2019. Corynebacterium diphtheriae: diphtheria toxin, the tox operon, and its regulation by Fe^2+^ activation of apo-DtxR. Microbiol Spectr 7. doi:10.1128/microbiolspec.GPP3-0063-2019 PMC8713076 31267892

[50] Martinez-Pastor M, Lancaster WA, Tonner PD, Adams MWW, Schmid AK. 2017. A transcription network of interlocking positive feedback loops maintains intracellular iron balance in Archaea. Nucleic Acids Res 45:9990–10001. doi:10.1093/nar/gkx662 28973467 PMC5737653

[51] Leyn SA, Rodionov DA. 2015. Comparative genomics of DtxR family regulons for metal homeostasis in Archaea. J Bacteriol 197:451–458. doi:10.1128/JB.02386-14 25404694 PMC4285986

[52] Schmid AK, Pan M, Sharma K, Baliga NS. 2011. Two transcription factors are necessary for iron homeostasis in a salt-dwelling archaeon. Nucleic Acids Res 39:2519–2533. doi:10.1093/nar/gkq1211 21109526 PMC3074139

[53] Pfeifer F. 2015. Haloarchaea and the formation of gas vesicles. Life (Basel) 5:385–402. doi:10.3390/life5010385 25648404 PMC4390858

[54] Scheuch S, Marschaus L, Sartorius-Neef S, Pfeifer F. 2008. Regulation of gvp genes encoding gas vesicle proteins in halophilic archaea. Arch Microbiol 190:333–339. doi:10.1007/s00203-008-0362-x 18385982

[55] Hechler T, Pfeifer F. 2009. Anaerobiosis inhibits gas vesicle formation in halophilic Archaea. Mol Microbiol 71:132–145. doi:10.1111/j.1365-2958.2008.06517.x 19007418

[56] Shand RF, Betlach MC. 1991. Expression of the bop gene cluster of Halobacterium halobium is induced by low oxygen tension and by light. J Bacteriol 173:4692–4699. doi:10.1128/jb.173.15.4692-4699.1991 1856168 PMC208146

[57] Grivard A, Goubet I, Duarte Filho LM de S, Thiéry V, Chevalier S, de Oliveira-Junior RG, El Aouad N, Guedes da Silva Almeida JR, Sitarek P, Quintans-Junior LJ, Grougnet R, Agogué H, Picot L. 2022. Archaea carotenoids: natural pigments with unexplored innovative potential. Mar Drugs 20:524. doi:10.3390/md20080524 36005527 PMC9410494

[58] Oren A. 2013. Life at high salt and low oxygen: how do the halobacteriaceae cope with low oxygen concentrations in their environment?, p 531–548. In Seckbach Joseph, Oren Aharon, Stan-Lotter Helga (ed), Polyextremophiles. life under multiple forms of stress. Vol. 27.

[59] Baliga NS, Kennedy SP, Ng WV, Hood L, DasSarma S. 2001. Genomic and genetic dissection of an archaeal regulon. Proc Natl Acad Sci U S A 98:2521–2525. doi:10.1073/pnas.051632498 11226271 PMC30170

[60] Müller JA, DasSarma S. 2005. Genomic analysis of anaerobic respiration in the archaeon Halobacterium sp. strain NRC-1: dimethyl sulfoxide and trimethylamine N-oxide as terminal electron acceptors. J Bacteriol 187:1659–1667. doi:10.1128/JB.187.5.1659-1667.2005 15716436 PMC1064022

[61] Thomas PD, Campbell MJ, Kejariwal A, Mi H, Karlak B, Daverman R, Diemer K, Muruganujan A, Narechania A. 2003. PANTHER: a library of protein families and subfamilies indexed by function. Genome Res 13:2129–2141. doi:10.1101/gr.772403 12952881 PMC403709

[62] Huergo LF, Chandra G, Merrick M. 2013. PII signal transduction proteins: nitrogen regulation and beyond. FEMS Microbiol Rev 37:251–283. doi:10.1111/j.1574-6976.2012.00351.x 22861350

[63] Forchhammer K, Selim KA, Huergo LF. 2022. New views on PII signaling: from nitrogen sensing to global metabolic control. Trends Microbiol 30:722–735. doi:10.1016/j.tim.2021.12.014 35067429

[64] Pedro-Roig L, Camacho M, Bonete MJ. 2011. In vitro proof of direct regulation of glutamine synthetase by GlnK proteins in the extreme halophilic archaeon Haloferax mediterranei. Biochem Soc Trans 39:259–262. doi:10.1042/BST0390259 21265784

[65] Esclapez J, Pire C, Camacho M, Bautista V, Martínez-Espinosa RM, Zafrilla B, Vegara A, Alcaraz LA, Bonete MJ. 2015. Transcriptional profiles of Haloferax mediterranei based on nitrogen availability. J Biotechnol 193:100–107. doi:10.1016/j.jbiotec.2014.11.018 25435380

[66] Rodríguez-Herrero V, Payá G, Bautista V, Vegara A, Cortés-Molina M, Camacho M, Esclapez J, Bonete MJ. 2020. Essentiality of the glnA gene in Haloferax mediterranei: gene conversion and transcriptional analysis. Extremophiles 24:433–446. doi:10.1007/s00792-020-01169-x 32296946

[67] Rodríguez-Herrero V, Peris A, Camacho M, Bautista V, Esclapez J, Bonete MJ. 2021. Novel glutamate–putrescine ligase activity in Haloferax mediterranei: a new function for glnA-2 gene. Biomolecules 11:1156. doi:10.3390/biom11081156 34439822 PMC8394153

[68] Yoshida M, Kashiwagi K, Shigemasa A, Taniguchi S, Yamamoto K, Makinoshima H, Ishihama A, Igarashi K. 2004. A unifying model for the role of polyamines in bacterial cell growth, the polyamine modulon. J Biol Chem 279:46008–46013. doi:10.1074/jbc.M404393200 15326188

[69] Lemmens L, Maklad HR, Bervoets I, Peeters E. 2019. Transcription regulators in Archaea: homologies and differences with bacterial regulators. J Mol Biol 431:4132–4146. doi:10.1016/j.jmb.2019.05.045 31195017

[70] Martinez-Pastor M, Tonner PD, Darnell CL, Schmid AK. 2017. Transcriptional regulation in Archaea: from individual genes to global regulatory networks. Annu Rev Genet 51:143–170. doi:10.1146/annurev-genet-120116-023413 29178818

[71] Karr EA. 2010. The methanogen-specific transcription factor MsvR regulates the fpaA-rlp-rub oxidative stress operon adjacent to msvR in Methanothermobacter thermautotrophicus. J Bacteriol 192:5914–5922. doi:10.1128/JB.00816-10 20851905 PMC2976444

[72] Cheng Y, Lyu M, Yang R, Wen Y, Song Y, Li J, Chen Z. 2020. SufR, a [4Fe-4S] cluster-containing transcription factor, represses the sufRBDCSU operon in Streptomyces avermitilis iron-sulfur cluster assembly. Appl Environ Microbiol 86:e01523-20. doi:10.1128/AEM.01523-20 32680866 PMC7480368

[73] Brinkman AB, Ettema TJG, de Vos WM, van der Oost J. 2003. The Lrp family of transcriptional regulators. Mol Microbiol 48:287–294. doi:10.1046/j.1365-2958.2003.03442.x 12675791

[74] Matarredona L, Camacho M, García-Bonete MJ, Esquerra B, Zafrilla B, Esclapez J, Bonete MJ. 2021. Analysis of Haloferax mediterranei Lrp transcriptional regulator. Genes (Basel) 12:802. doi:10.3390/genes12060802 34070366 PMC8229911

[75] Pastor-Soler S, Camacho M, Bautista V, Bonete MJ, Esclapez J. 2021. Towards the elucidation of assimilative nasabc operon transcriptional regulation in Haloferax mediterranei. Genes (Basel) 12:619. doi:10.3390/genes12050619 33921943 PMC8143581

[76] Zhang RG, Kim Y, Skarina T, Beasley S, Laskowski R, Arrowsmith C, Edwards A, Joachimiak A, Savchenko A. 2002. Crystal structure of Thermotoga maritima 0065, a member of the IclR transcriptional factor family. J Biol Chem 277:19183–19190. doi:10.1074/jbc.M112171200 11877432 PMC2792004

[77] Johnsen U, Sutter JM, Schulz AC, Tästensen JB, Schönheit P. 2015. XacR - A novel transcriptional regulator of D-xylose and L-arabinose catabolism in the haloarchaeon Haloferax volcanii. Environ Microbiol 17:1663–1676. doi:10.1111/1462-2920.12603 25141768

[78] Molina-Henares AJ, Krell T, Eugenia Guazzaroni M, Segura A, Ramos JL. 2006. Members of the IclR family of bacterial transcriptional regulators function as activators and/or repressors. FEMS Microbiol Rev 30:157–186. doi:10.1111/j.1574-6976.2005.00008.x 16472303

[79] Zhou Y, Huang H, Zhou P, Xie J. 2012. Molecular mechanisms underlying the function diversity of transcriptional factor IclR family. Cell Signal 24:1270–1275. doi:10.1016/j.cellsig.2012.02.008 22382436

[80] Paula FS, Chin JP, Schnürer A, Müller B, Manesiotis P, Waters N, Macintosh KA, Quinn JP, Connolly J, Abram F, McGrath JW, O’Flaherty V. 2019. The potential for polyphosphate metabolism in Archaea and anaerobic polyphosphate formation in Methanosarcina mazei. Sci Rep 9:17101. doi:10.1038/s41598-019-53168-4 31745137 PMC6864096

[81] Wende A, Furtwängler K, Oesterhelt D. 2009. Phosphate-dependent behavior of the archaeon Halobacterium salinarum strain R1. J Bacteriol 191:3852–3860. doi:10.1128/JB.01642-08 19363117 PMC2698381

[82] Bailey TL, Johnson J, Grant CE, Noble WS. 2015. The MEME suite. Nucleic Acids Res 43:W39–W49. doi:10.1093/nar/gkv416 25953851 PMC4489269

[83] Philippot L. 2002. Denitrifying genes in bacterial and Archaeal genomes. Biochimica et Biophysica Acta (BBA) - Gene Structure and Expression 1577:355–376. doi:10.1016/S0167-4781(02)00420-7 12359326

[84] Mushegian AR, Koonin EV. 1996. Gene order is not conserved in bacterial evolution. Trends in Genetics 12:289–290. doi:10.1016/0168-9525(96)20006-X 8783936

[85] Lathe WC, Snel B, Bork P. 2000. Gene context conservation of a higher order than operons. Trends Biochem Sci 25:474–479. doi:10.1016/s0968-0004(00)01663-7 11050428

[86] Llorca MG, Martínez-Espinosa RM. 2022. Assessment of Haloferax mediterranei genome in search of copper-molecular machinery with potential applications for bioremediation. Front Microbiol 13:895296. doi:10.3389/fmicb.2022.895296 35783429 PMC9240420

[87] Srivastava P, Kowshik M. 2013. Mechanisms of metal resistance and homeostasis in haloarchaea. Archaea 2013:732864. doi:10.1155/2013/732864 23533331 PMC3600143

[88] Bini E. 2010. Archaeal transformation of metals in the environment. FEMS Microbiol Ecol 73:1–16. doi:10.1111/j.1574-6941.2010.00876.x 20455933

[89] Miralles-Robledillo JM, Torregrosa-Crespo J, Martínez-Espinosa RM, Pire C. 2019. DMSO reductase family: phylogenetics and applications of extremophiles. Int J Mol Sci 20:3349. doi:10.3390/ijms20133349 31288391 PMC6650914

[90] Wang G, Kennedy SP, Fasiludeen S, Rensing C, DasSarma S. 2004. Arsenic resistance in Halobacterium sp. strain NRC-1 examined by using an improved gene knockout system. J Bacteriol 186:3187–3194. doi:10.1128/JB.186.10.3187-3194.2004 15126481 PMC400623

[91] Petrarca P, Ammendola S, Pasquali P, Battistoni A. 2010. The zur-regulated ZinT protein is an auxiliary component of the high-affinity ZnuABC zinc transporter that facilitates metal recruitment during severe zinc shortage. J Bacteriol 192:1553–1564. doi:10.1128/JB.01310-09 20097857 PMC2832539

[92] Märkle P, Maier LK, Maaß S, Hirschfeld C, Bartel J, Becher D, Voß B, Marchfelder A. 2021. A small RNA is linking CRISPR–Cas and zinc transport. Front Mol Biosci 8:640440. doi:10.3389/fmolb.2021.640440 34055875 PMC8155600

[93] Payá G, Bautista V, Camacho M, Bonete MJ, Esclapez J. 2020. New proposal of nitrogen metabolism regulation by small RNAs in the extreme halophilic archaeon Haloferax mediterranei. Mol Genet Genomics 295:775–785. doi:10.1007/s00438-020-01659-9 32170429

[94] Kaur K, Sharma A, Capalash N, Sharma P. 2019. Multicopper oxidases: biocatalysts in microbial pathogenesis and stress management. Microbiol Res 222:1–13. doi:10.1016/j.micres.2019.02.007 30928025

[95] Banci L, Bertini I, Cavallaro G, Ciofi‐Baffoni S. 2011. Seeking the determinants of the elusive functions of Sco proteins. The FEBS Journal 278:2244–2262. doi:10.1111/j.1742-4658.2011.08141.x 21518258

[96] Pauleta SR, Carreira C, Moura I. 2017. CHAPTER 7: insights into nitrous oxide reductase, p 141–169. In Moura Isabel, Moura José JG, Pauleta Sofia R, Maia Luisa B (ed), Metalloenzymes in denitrification: applications and environmental impacts

[97] Wunsch P, Herb M, Wieland H, Schiek UM, Zumft WG. 2003. Requirements for CuA and Cu-S center assembly of nitrous oxide reductase deduced from complete periplasmic enzyme maturation in the non denitrifier Pseudomonas putida. J Bacteriol 185:887–896. doi:10.1128/JB.185.3.887-896.2003 12533464 PMC142834

[98] Pauleta S, Moura I. 2017. Assembly of CuZ and CuA in nitrous oxide reductase. Encyclopedia of Inorganic and Bioinorganic Chemistry:1–11. doi:10.1002/9781119951438

[99] Hantke K. 2001. Iron and metal regulation in Bacteria. Curr Opin Microbiol 4:172–177. doi:10.1016/s1369-5274(00)00184-3 11282473

[100] Wong DK, Lee B-Y, Horwitz MA, Gibson BW. 1999. Identification of fur, aconitase, and other proteins expressed by Mycobacterium tuberculosis under conditions of low and high concentrations of iron by combined two-dimensional gel electrophoresis and mass spectrometry. Infect Immun 67:327–336. doi:10.1128/IAI.67.1.327-336.1999 9864233 PMC96314

[101] Self WT, Grunden AM, Hasona A, Shanmugam KT. 2001. Molybdate transport. Res Microbiol 152:311–321. doi:10.1016/s0923-2508(01)01202-5 11421278

[102] Andrews S. 2010. FastQC a quality control tool for high throughput sequence data. Available from: https://www.bioinformatics.babraham.ac.uk/projects/fastqc. Retrieved 26 May 2022.

[103] Bolger AM, Lohse M, Usadel B. 2014. Trimmomatic: a flexible trimmer for Illumina sequence data. Bioinformatics 30:2114–2120. doi:10.1093/bioinformatics/btu170 24695404 PMC4103590

[104] Langmead B, Salzberg SL. 2012. Fast gapped-read alignment with Bowtie 2. Nat Methods 9:357–359. doi:10.1038/nmeth.1923 22388286 PMC3322381

[105] García-Alcalde F, Okonechnikov K, Carbonell J, Cruz LM, Götz S, Tarazona S, Dopazo J, Meyer TF, Conesa A. 2012. Qualimap: evaluating next-generation sequencing alignment data. Bioinformatics 28:2678–2679. doi:10.1093/bioinformatics/bts503 22914218

[106] Liao Y, Smyth GK, Shi W. 2014. featureCounts: an efficient general purpose program for assigning sequence reads to genomic features. Bioinformatics 30:923–930. doi:10.1093/bioinformatics/btt656 24227677

[107] Subramanian A, Tamayo P, Mootha VK, Mukherjee S, Ebert BL, Gillette MA, Paulovich A, Pomeroy SL, Golub TR, Lander ES, Mesirov JP. 2005. Gene set enrichment analysis: a knowledge-based approach for interpreting genome-wide expression profiles. Proc Natl Acad Sci U S A 102:15545–15550. doi:10.1073/pnas.0506580102 16199517 PMC1239896

[108] Love MI, Huber W, Anders S. 2014. Moderated estimation of fold change and dispersion for RNA-seq data with DESeq2. Genome Biol 15:550. doi:10.1186/s13059-014-0550-8 25516281 PMC4302049

[109] Yu G, Wang LG, Han Y, He QY. 2012. ClusterProfiler: an R package for comparing biological themes among gene clusters. OMICS 16:284–287. doi:10.1089/omi.2011.0118 22455463 PMC3339379

[110] Thorvaldsdóttir H, Robinson JT, Mesirov JP. 2013. Integrative Genomics Viewer (IGV): high-performance genomics data visualization and exploration. Brief Bioinform 14:178–192. doi:10.1093/bib/bbs017 22517427 PMC3603213

[111] Bateman A, Martin M-J, Orchard S, Magrane M, Agivetova R, Ahmad S, Alpi E, Bowler-Barnett EH, Britto R, Bursteinas B. 2021. UniProt: the universal protein knowledgebase in 2021. Nucleic Acids Res 49:D480–D489. doi:10.1093/nar/gkaa1100 33237286 PMC7778908

[112] Tamura K, Stecher G, Kumar S. 2021. MEGA11: molecular evolutionary genetics analysis version 11. Mol Biol Evol 38:3022–3027. doi:10.1093/molbev/msab120 33892491 PMC8233496

