# Supplementary material for: Transcriptomic profiling of haloarchaeal denitrification through RNA-Seq analysis
Source: Appl Environ Microbiol. 2024 May 30;90(6):e00571-24. doi: 10.1128/aem.00571-24 (PMC11218638; doi:10.1128/aem.00571-24)
Supplement: Supplemental material — Tables S1 to S7. [file aem.00571-24-s0001.docx]

**SUPPLEMENTARY MATERIAL:**

**SUPPLEMENTARY TABLE 1.** Gene Set Enrichment Analysis output.

| **ID** | **Description** | **setSize** | **NES** | **pvalue** | **p.adjust** | **qvalues** | **rank** | **leading_edge** | **core_enrichment** |
| --- | --- | --- | --- | --- | --- | --- | --- | --- | --- |
| hme00910 | Nitrogen metabolism | 14 | 2,17059E+14 | 1,56691E+14 | 1,56691E+14 | 5,04097E+14 | 192 | tags=57%, list=8%, signal=53% | E6P09_00775, E6P09_00750, E6P09_17445, E6P09_17440, E6P09_15580, E6P09_17380, E6P09_07245, E6P09_02095 |
| hme00240 | Pyrimidine metabolism | 26 | -1,978E+14 | 3,16857E+14 | 3,16857E+14 | 5,04097E+14 | 585 | tags=69%, list=25%, signal=53% | E6P09_14945, E6P09_14150, E6P09_15840, E6P09_13765, E6P09_10630, E6P09_01450, E6P09_14210, E6P09_11230, E6P09_16690, E6P09_13570, E6P09_01960, E6P09_02795, E6P09_02235, E6P09_12730, E6P09_13630, E6P09_04680, E6P09_12360, E6P09_13020 |
| hme00190 | Oxidative phosphorylation | 33 | -2,18916E+14 | 3,48554E+14 | 3,48554E+14 | 5,04097E+14 | 520 | tags=70%, list=22%, signal=55% | E6P09_07965, E6P09_07975, E6P09_07930, E6P09_04615, E6P09_04575, E6P09_07945, E6P09_07935, E6P09_07950, E6P09_07940, E6P09_07960, E6P09_04605, E6P09_04585, E6P09_04570, E6P09_04600, E6P09_08090, E6P09_04590, E6P09_04580, E6P09_04595, E6P09_07955, E6P09_07925, E6P09_11225, E6P09_08765, E6P09_08095 |
| hme00230 | Purine metabolism | 38 | -2,22736E+14 | 3,6049E+14 | 3,6049E+14 | 5,04097E+14 | 297 | tags=71%, list=13%, signal=63% | E6P09_13680, E6P09_01235, E6P09_00195, E6P09_15840, E6P09_10415, E6P09_06895, E6P09_06135, E6P09_14200, E6P09_13115, E6P09_16690, E6P09_00460, E6P09_08265, E6P09_08490, E6P09_02855, E6P09_00455, E6P09_07920, E6P09_13565, E6P09_00435, E6P09_08720, E6P09_11130, E6P09_11775, E6P09_07915, E6P09_08395, E6P09_04685, E6P09_03145, E6P09_13020, E6P09_09420 |
| hme03010 | Ribosome | 51 | -3,90567E+14 | 4,05022E+14 | 4,05022E+14 | 5,04097E+14 | 270 | tags=94%, list=11%, signal=85% | E6P09_06170, E6P09_12860, E6P09_03720, E6P09_13840, E6P09_12810, E6P09_13885, E6P09_13915, E6P09_13920, E6P09_13845, E6P09_13925, E6P09_12805, E6P09_13900, E6P09_12840, E6P09_13905, E6P09_13890, E6P09_12945, E6P09_06340, E6P09_12835, E6P09_14885, E6P09_13940, E6P09_12830, E6P09_12940, E6P09_05090, E6P09_12815, E6P09_13895, E6P09_13930, E6P09_12935, E6P09_13870, E6P09_13945, E6P09_13935, E6P09_13850, E6P09_13950, E6P09_05320, E6P09_12975, E6P09_13875, E6P09_06345, E6P09_13860, E6P09_04785, E6P09_13865, E6P09_13855, E6P09_03715, E6P09_08800, E6P09_12930, E6P09_08815, E6P09_02285, E6P09_05710, E6P09_13025, E6P09_13035 |
| hme03020 | RNA polymerase | 8 | -2,10318E+14 | 4,98753E+14 | 4,98753E+14 | 5,04097E+14 | 237 | tags=75%, list=10%, signal=68% | E6P09_01740, E6P09_12820, E6P09_12845, E6P09_12850, E6P09_12980, E6P09_01745 |
| hme01240 | Biosynthesis of cofactors | 84 | -1,82737E+14 | 5,1573E+14 | 5,1573E+14 | 5,04097E+14 | 723 | tags=60%, list=30%, signal=43% | E6P09_00055, E6P09_10115, E6P09_02705, E6P09_02685, E6P09_05920, E6P09_10635, E6P09_05915, E6P09_00060, E6P09_11740, E6P09_18600, E6P09_12210, E6P09_10685, E6P09_07065, E6P09_14945, E6P09_03525, E6P09_05075, E6P09_14150, E6P09_18595, E6P09_00065, E6P09_18585, E6P09_11825, E6P09_18590, E6P09_13765, E6P09_04660, E6P09_15870, E6P09_06385, E6P09_10700, E6P09_18335, E6P09_10630, E6P09_15855, E6P09_02180, E6P09_06895, E6P09_03530, E6P09_06235, E6P09_08090, E6P09_06930, E6P09_18340, E6P09_14200, E6P09_02230, E6P09_13570, E6P09_01960, E6P09_02235, E6P09_06215, E6P09_10705, E6P09_04680, E6P09_08720, E6P09_06230, E6P09_12495, E6P09_10670, E6P09_13020 |
| hme00360 | Phenylalanine metabolism | 13 | 1,91725E+14 | 1,74161E+14 | 1,74161E+14 | 1,48953E+14 | 383 | tags=69%, list=16%, signal=58% | E6P09_16060, E6P09_16055, E6P09_16065, E6P09_16130, E6P09_05945, E6P09_10585, E6P09_16090, E6P09_16135, E6P09_15355 |
| hme01120 | Microbial metabolism in diverse environments | 131 | 1,60871E+14 | 3,27026E+14 | 3,27026E+14 | 2,48617E+14 | 471 | tags=37%, list=20%, signal=31% | E6P09_00775, E6P09_00750, E6P09_17445, E6P09_17440, E6P09_17735, E6P09_15580, E6P09_15575, E6P09_15465, E6P09_16930, E6P09_16060, E6P09_05600, E6P09_10845, E6P09_17380, E6P09_16055, E6P09_09220, E6P09_16065, E6P09_15290, E6P09_18965, E6P09_15365, E6P09_07245, E6P09_02095, E6P09_18455, E6P09_15690, E6P09_00865, E6P09_18490, E6P09_16080, E6P09_04165, E6P09_10830, E6P09_03210, E6P09_10585, E6P09_16090, E6P09_07375, E6P09_08565, E6P09_07880, E6P09_08570, E6P09_10850, E6P09_16135, E6P09_08560, E6P09_16990, E6P09_14840, E6P09_03130, E6P09_12460, E6P09_05245, E6P09_04805, E6P09_01550, E6P09_15370, E6P09_16150, E6P09_16120 |
| hme00300 | Lysine biosynthesis | 13 | 1,80944E+14 | 4,11653E+14 | 4,11653E+14 | 2,81657E+13 | 735 | tags=92%, list=31%, signal=64% | E6P09_08565, E6P09_08570, E6P09_08560, E6P09_03130, E6P09_03325, E6P09_04805, E6P09_03320, E6P09_03315, E6P09_08555, E6P09_08550, E6P09_03310, E6P09_03305 |
| hme02060 | Phosphotransferase system (PTS) | 7 | 1,75424E+14 | 5,11683E+14 | 5,11683E+14 | 3,18272E+14 | 439 | tags=86%, list=18%, signal=70% | E6P09_10845, E6P09_10840, E6P09_10830, E6P09_10850, E6P09_10835, E6P09_10855 |
| hme03060 | Protein export | 12 | -1,78356E+14 | 8,5699E+14 | 8,5699E+14 | 4,88635E+14 | 656 | tags=75%, list=28%, signal=55% | E6P09_04030, E6P09_03740, E6P09_01355, E6P09_06430, E6P09_01360, E6P09_13670, E6P09_08605, E6P09_08890, E6P09_13955 |
| hme00970 | Aminoacyl-tRNA biosynthesis | 25 | -1,68998E+14 | 1,47567E+13 | 1,47567E+13 | 7,76667E+14 | 836 | tags=76%, list=35%, signal=50% | E6P09_13090, E6P09_04150, E6P09_11910, E6P09_11075, E6P09_04635, E6P09_06280, E6P09_08690, E6P09_01625, E6P09_03370, E6P09_01955, E6P09_06245, E6P09_02620, E6P09_14135, E6P09_08300, E6P09_02635, E6P09_02630, E6P09_05155, E6P09_08295, E6P09_07250 |
| hme03030 | DNA replication | 17 | -1,71075E+14 | 1,70915E+14 | 1,70915E+14 | 8,35301E+14 | 766 | tags=76%, list=32%, signal=52% | E6P09_07650, E6P09_01345, E6P09_12450, E6P09_03980, E6P09_07160, E6P09_12890, E6P09_06495, E6P09_14610, E6P09_03870, E6P09_11165, E6P09_03965, E6P09_03110, E6P09_13235 |
| hme02024 | Quorum sensing | 40 | 1,57691E+14 | 2,31118E+14 | 2,31118E+14 | 1,05422E+14 | 553 | tags=48%, list=23%, signal=37% | E6P09_14700, E6P09_14550, E6P09_14560, E6P09_14555, E6P09_14545, E6P09_18950, E6P09_14470, E6P09_09030, E6P09_08410, E6P09_09045, E6P09_12710, E6P09_14540, E6P09_08405, E6P09_08415, E6P09_16100, E6P09_12700, E6P09_16105, E6P09_08420, E6P09_02535 |
| hme00071 | Fatty acid degradation | 13 | 1,62109E+14 | 2,56491E+13 | 2,56491E+13 | 1,09684E+14 | 264 | tags=54%, list=11%, signal=48% | E6P09_15465, E6P09_10475, E6P09_15290, E6P09_18965, E6P09_16080, E6P09_04165, E6P09_18950 |
| hme00270 | Cysteine and methionine metabolism | 23 | 1,58289E+14 | 2,80816E+13 | 2,80816E+13 | 1,10571E+14 | 425 | tags=43%, list=18%, signal=36% | E6P09_17735, E6P09_03000, E6P09_03010, E6P09_03005, E6P09_13005, E6P09_13010, E6P09_03210, E6P09_15355, E6P09_03130, E6P09_04805 |
| hme00920 | Sulfur metabolism | 11 | 1,61398E+14 | 2,90886E+14 | 2,90886E+14 | 1,10571E+14 | 266 | tags=36%, list=11%, signal=32% | E6P09_17735, E6P09_03670, E6P09_03005, E6P09_03210 |
| hme00670 | One carbon pool by folate | 8 | -1,65179E+14 | 3,09227E+14 | 3,09227E+14 | 1,11356E+14 | 798 | tags=88%, list=34%, signal=58% | E6P09_12215, E6P09_00445, E6P09_01590, E6P09_12210, E6P09_14745, E6P09_08490, E6P09_12495 |
| hme03070 | Bacterial secretion system | 8 | -1,62375E+14 | 3,4414E+14 | 3,4414E+14 | 1,17732E+14 | 656 | tags=75%, list=28%, signal=54% | E6P09_04030, E6P09_03740, E6P09_01355, E6P09_01360, E6P09_08890, E6P09_13955 |
| hme00260 | Glycine, serine and threonine metabolism | 24 | -1,56211E+14 | 3,88319E+14 | 3,88319E+14 | 1,25115E+14 | 232 | tags=17%, list=10%, signal=15% | E6P09_14755, E6P09_14760, E6P09_18550, E6P09_18555 |
| hme00380 | Tryptophan metabolism | 13 | 1,56121E+14 | 4,06903E+14 | 4,06903E+14 | 1,25115E+14 | 274 | tags=46%, list=12%, signal=41% | E6P09_15465, E6P09_15290, E6P09_18965, E6P09_16080, E6P09_04165, E6P09_03135 |
| hme00625 | Chloroalkane and chloroalkene degradation | 3 | 1,4619E+13 | 4,20578E+14 | 4,20578E+14 | 1,25115E+14 | 397 | tags=100%, list=17%, signal=83% | E6P09_18965, E6P09_16080, E6P09_14840 |
| hme01230 | Biosynthesis of amino acids | 73 | 1,42463E+14 | 4,82811E+14 | 4,82811E+14 | 1,33323E+14 | 568 | tags=44%, list=24%, signal=34% | E6P09_17735, E6P09_03005, E6P09_05600, E6P09_13005, E6P09_13010, E6P09_15395, E6P09_14465, E6P09_02080, E6P09_13430, E6P09_05945, E6P09_14460, E6P09_14470, E6P09_08565, E6P09_06765, E6P09_10895, E6P09_06120, E6P09_08570, E6P09_15355, E6P09_08560, E6P09_10890, E6P09_16990, E6P09_03130, E6P09_05245, E6P09_03325, E6P09_04805, E6P09_03320, E6P09_03315, E6P09_10870, E6P09_11325, E6P09_08555, E6P09_06775, E6P09_08550 |
| hme02020 | Two-component system | 20 | 1,5199E+14 | 4,87141E+13 | 4,87141E+13 | 1,33323E+14 | 172 | tags=25%, list=7%, signal=23% | E6P09_17445, E6P09_17440, E6P09_15465, E6P09_03215, E6P09_15290 |

**SUPPLEMENTARY TABLE 2.** Nitrate reductase gene cluster. Each gene is shown together with its product and its log_2_FC value (oxic *vs.* denitrifying conditions).

| **NCBI locus tag** | **Gene product** | **log_2_FC** |
| --- | --- | --- |
| E6P09_17405 | pseudo | 1.99 |
| E6P09_17410 | molybdopterin-dependent oxidoreductase | 2.46 |
| E6P09_17415 | P-loop NTPase | 3.60 |
| E6P09_17420 | HEAT repeat domain-containing protein | 4.23 |
| E6P09_17425 | hypothetical protein | 4.53 |
| E6P09_17430 | molecular chaperone TorD family protein (NarJ) | 4.74 |
| E6P09_17435 | DMSO reductase family type II enzyme. heme b subunit | 5.18 |
| E6P09_17440 | nitrate reductase subunit beta (NarH) | 4.88 |
| E6P09_17445 | nitrate reductase subunit Alpha (NarG) | 5.34 |
| E6P09_17450 | hypothetical protein | 6.49 |
| E6P09_17455 | cytochrome bc complex cytochrome b subunit (NarC) | 5.08 |
| E6P09_17460 | Rieske 2Fe-2S domain-containing protein (NarB) | 4.73 |
| E6P09_17465 | hypothetical protein | 4.24 |

**SUPPLEMENTARY TABLE 3.** Nitrite and nitric oxide reductase gene cluster. Each gene is shown together with its product and its log_2_FC value (oxic *vs.* denitrifying conditions).

| **NCBI locus tag** | **Gene product** | **log_2_FC** |
| --- | --- | --- |
| E6P09_00735 | helix-turn-helix domain-containing protein (Bacterioopsin activator) | 3.50 |
| E6P09_00740 | halocyanin domain-containing protein | 2.95 |
| E6P09_00745 | CGCGG family rSAM-modified RiPP protein | 4.30 |
| E6P09_00750 | cbb3-type cytochrome c oxidase subunit I (Nor) | 5.73 |
| E6P09_00755 | IS1595 family transposase | 0.85 |
| E6P09_00760 | halocyanin domain-containing protein | 4.16 |
| E6P09_00765 | TIGR04053 family radical SAM/SPASM domain-containing protein | 2.78 |
| E6P09_00770 | DUF2249 domain-containing protein | 8.65 |
| E6P09_00775 | nitrite reductase. copper-containing (NirK) | 8.06 |
| E6P09_00780 | hypothetical protein | 7.93 |
| E6P09_00785 | cupin domain-containing protein | 8.10 |
| E6P09_00790 | pseudo | 5.60 |
| E6P09_00795 | DUF2249 domain-containing protein | 3.24 |

**SUPPLEMENTARY TABLE 4.** Nitrous reductase gene cluster. Each gene is shown together with its product and its log_2_FC value (oxic *vs.* denitrifying conditions).

| **NCBI locus tag** | **Gene product** | **log_2_FC** |
| --- | --- | --- |
| E6P09_18750 | nitrous oxide reductase accessory protein (NosL) | 2.21 |
| E6P09_18755 | ABC transporter permease (NosY) | 2.28 |
| E6P09_18760 | ABC transporter ATP-binding protein (NosF) | 2.41 |
| E6P09_17370 | nitrous oxide reductase family maturation protein (NosD) | 2.48 |
| E6P09_17375 | hypothetical protein | 2.72 |
| E6P09_17380 | nitrous-oxide reductase (NosZ) | 2.82 |
| E6P09_17385 | copper-binding protein (Azurin) | 2.65 |
| E6P09_17390 | hypothetical protein | 6.50 |
| E6P09_17395 | DUF2249 domain-containing protein | 7.09 |

| **Location** | **start** | **stop** | **p-value** | **motif** | **Closest locus** | **log_2_FC** | **Gene product** | **Closest locus*** | **log_2_FC** | **Gene product** |
| --- | --- | --- | --- | --- | --- | --- | --- | --- | --- | --- |
| Chr | 143508 | 143519 | 4.05E-05 | CGAATGTTTTTG | E6P09_00735 | 3,495941236 | helix-turn-helix domain-containing protein | E6P09_00740 | 2,946013774 | **halocyanin domain-containing protein** |
| Chr | 145214 | 145225 | 5.62E-08 | CGAACATGTTCG | E6P09_00745 | 4,296554737 | CGCGG family rSAM-modified RiPP protein | E6P09_00750 | 5,730165181 | **cbb3-type cytochrome c oxidase subunit I (nor)** |
| Chr | 149575 | 149586 | 5.62E-08 | CGAACATGTTCG | E6P09_00760 | 4,156256718 | halocyanin domain-containing protein |  |  |  |
| Chr | 150859 | 150870 | 8.22E-07 | CGAATATATTCG | E6P09_00770 | 8,651566783 | DUF2249 domain-containing protein | E6P09_00765 | 2,782006364 | TIGR04053 family radical SAM/SPASM domain-containing protein |
| Chr | 151382 | 151393 | 3.76E-07 | CGAATATGTTCG | E6P09_00775 | 8,060896979 | **copper-containing nitrite reductase (nirK)** |  |  |  |
| Chr | 152650 | 152661 | 7.04E-05 | CGGACATGTTCA | E6P09_00780 | 7,9306392 | hypothetical protein |  |  |  |
| Chr | 153713 | 153724 | 6.02E-05 | CGAACCGGTTCG | E6P09_00785 | 8,099891077 | cupin domain-containing protein |  |  |  |
| Chr | 155152 | 155163 | 3.76E-07 | CGAATATGTTCG | E6P09_00790 | 5,603317932 | **hypothetical protein (pseudogene)** |  |  |  |
| Chr | 1413849 | 1413860 | 8.94E-05 | CGAACTTATTAG | E6P09_07160 | -0,818897971 | DNA polymerase | E6P09_07155 | 2,169901285 | hypothetical protein |
| Chr | 1773828 | 1773839 | 7.13E-06 | CGACCATGGTCG | E6P09_09025 | 2,085083106 | ABC transporter substrate-binding protein | E6P09_09020 | 0,350911756 | proline dehydrogenase family protein |
| Chr | 2174636 | 2174647 | 2.95E-05 | CGCATATGTTTG | E6P09_11130 | -2,015565904 | phosphoribosylformylglycinamidine cyclo-ligase |  |  |  |
| Chr | 2306535 | 2306546 | 1.14E-05 | CGAATTTGTTCG | E6P09_11805 | 4,157309387 | group 1 truncated haemoglobin |  |  |  |
| Chr | 2886061 | 2886072 | 7.83E-05 | TGAACATCGTCG | E6P09_14825 | 2,648768069 | CBS domain-containing protein | E6P09_14815 | 1,79067704 | hypothetical protein |
| Chr | 2899621 | 2899632 | 3.46E-05 | CGAATATAGCCG | E6P09_14875 | -2,999719206 | PstS family phosphate ABC transporter substrate-binding protein | E6P09_14880 | -2,357414388 | phosphate uptake regulator PhoU |
| pHME505 | 13418 | 13429 | 7.79E-05 | CGAACATACACG | E6P09_15175 | 4,967784021 | hypothetical protein |  |  |  |
| pHME505 | 46479 | 46490 | 1.77E-05 | CGAATATGGTAG | E6P09_15300 | 4,572993 | hypothetical protein | E6P09_15295 | 1,27532829 | ArsA family ATPase |
| pHME505 | 68603 | 68614 | 4.14E-05 | CGAATACGGTTG | E6P09_15400 | 0,985393696 | NAD-binding protein | E6P09_15395 | 2,268902919 | thiamine pyrophosphate-binding protein |
| pHME505 | 400264 | 400275 | 7.83E-05 | CAAACATCGTCG | E6P09_16935 | 2,725119783 | sugar kinase | E6P09_16930 |  | bifunctional 4-hydroxy-2-oxoglutarate aldolase/2-dehydro-3-deoxy-phosphogluconate aldolase |
| pHME322 | 4426 | 4437 | 5.62E-08 | CGAACATGTTCG | E6P09_17385 | 2,65072591 | **plastocyanin/azurin family copper-binding protein (nos operon)** | E6P09_17390 | 6,499319066 | hypothetical protein |
| pHME322 | 4588 | 4599 | 2.14E-06 | CGAAGATGTTCG | E6P09_17390 | 6,499319066 | hypothetical protein | E6P09_17385 | 2,65072591 | **plastocyanin/azurin family copper-binding protein (nos operon)** |
| pHME322 | 6870 | 6881 | 2.88E-06 | CGAACACGTTCG | E6P09_17400 | 7,006513655 | hypothetical protein |  |  |  |
| pHME322 | 20146 | 20157 | 1.73E-06 | CGAATATTGTTG | E6P09_17470 | -0,738589252 | TetR/AcrR family transcriptional regulator | E6P09_17465 | 4,241307897 | **hypothetical protein (nar operon)** |
| pHME322 | 63378 | 63389 | 6.02E-05 | CGAACCCGTTCG | E6P09_17615 | 4,7980081 | cupin domain-containing protein |  |  |  |
| pHME322 | 317695 | 317706 | 4.14E-05 | CGAATGTGGTTG | E6P09_18735 | 1,113224046 | ABC transporter ATP-binding protein | E6P09_18740 | 2,07976067 | hypothetical protein |
| pHME505 | 167496 | 167507 | 4.14E-05 | CGTATATGGTTG | E6P09_19800 | 2,656915179 | hypothetical protein |  |  |  |

**SUPPLEMENTARY TABLE 5.** Promoter regions that carry the regulatory motif CGAAYATDKTYG whose genes under its control displayed a differential expression under denitrifying conditions (log_2_FC < -2 or log_2_FC > +2 and padj < 0.05).

**SUPPLEMENTARY TABLE 6.** Expression changes of genes encoding the enzymes of the tricarboxylic acid cycle under denitrifying conditions (oxic *vs.* denitrifying conditions).

| **NCBI locus tag** | **Gene product** | **Log_2_FC** |
| --- | --- | --- |
| E6P09_05225 | citrate (Si)-synthase (CitZ) | -0,78 |
| E6P09_16990 | citrate synthase/methylcitrate synthase (GltA) | 1,40 |
| E6P09_05600 | aconitate hydratase (AcnA) | 2,89 |
| E6P09_01455 | aconitate hydratase (CitB) | -1,45 |
| E6P09_13710 | isocitrate dehydrogenase (NADP(+)) (Icd) | -2,35 |
| E6P09_02705 | dihydrolipoyl dehydrogenase (PdhD) | -0,78 |
| E6P09_07335 | 2-oxoacid:acceptor oxidoreductase subunit Alpha (KorA) | -1,99 |
| E6P09_07330 | 2-oxoacid:ferredoxin oxidoreductase subunit beta (KorB) | -1,57 |
| E6P09_14425 | succinate--CoA ligase subunit alpha (SucD) | -0,60 |
| E6P09_14420 | ADP-forming succinate--CoA ligase subunit beta (SucC) | -0,44 |
| E6P09_12660 | succinate dehydrogenase (SdhA) | -0,56 |
| E6P09_12655 | succinate dehydrogenase/fumarate reductase iron-sulfur subunit (SdhB) | -0,44 |
| E6P09_12645 | succinate dehydrogenase, cytochrome b556 subunit (SdhC) | -0,72 |
| E6P09_12650 | succinate dehydrogenase hydrophobic membrane anchor subunit (SdhD) | -0,57 |
| E6P09_12300 | class II fumarate hydratase (FumC) | -0,46 |
| E6P09_03065 | malate dehydrogenase (MdhA) | -0,18 |
| E6P09_02690 | pyruvate dehydrogenase (acetyl-transferring) E1 component subunit alpha (PdhA1) | -1,11 |
| E6P09_00365 | pyruvate dehydrogenase (acetyl-transferring) E1 component subunit alpha (OxdhA2) | -0,01 |
| E6P09_15365 | pyruvate dehydrogenase (acetyl-transferring) E1 component subunit alpha (PdhA2) | 2,39 |
| E6P09_02695 | alpha-ketoacid dehydrogenase subunit beta (PdhB1) | -1,14 |
| E6P09_15370 | alpha-ketoacid dehydrogenase subunit beta (PdhB2) | 1,21 |
| E6P09_02700 | 2-oxo acid dehydrogenase subunit E2 (PdhC) | -0,94 |
| E6P09_09880 | 2-oxoacid:acceptor oxidoreductase subunit Alpha (PorA) | 0,05 |
| E6P09_09875 | 2-ketoglutarate ferredoxin oxidoreductase subunit beta (PorB) | 0,14 |

**SUPPLEMENTARY TABLE 7.** List of primers used to check DNAse treatment.

| **Primer name** | **Primer sequence** | **Position** | **Amplicon size (pb)** |
| --- | --- | --- | --- |
| *pnarF* | GTCGCCTCCAAGCTTTCTTCCGC | 20220 - 20198 | 420 |
| *pnarR* | AAGATGGCCATGGTCTCTCGCCTCAT | 19801 - 19826 |  |
| *pnirF* | CGGGCAACAAGCTTCGGTCACG | 151234 - 151255 | 267 |
| *pnirR* | ACGTCCGTCCCATGGTTGTTGATAGCAT | 151500 - 151473 |  |
| *pnosF* | CGTTGACTAAGCTTCCGGGTGAGCAC | 4829 - 4804 | 501 |
| *pnosR* | CGTTCCCCATGGTATGTTTCCCTGCCAT | 4329 - 4359 |  |
